# Impacts of short-term feeding by spotted lanternfly (*Lycorma delicatula)* on ecophysiology of young hardwood trees in a common garden

**DOI:** 10.3389/finsc.2022.1080124

**Published:** 2022-12-07

**Authors:** Emily Lavely, Lidiia Iavorivska, Osariyekemwen Uyi, David M. Eissenstat, Brian Walsh, Edward J. Primka, Jeremy Harper, Kelli Hoover

**Affiliations:** ^1^ Department of Ecosystem Science and Management, Pennsylvania State University, University Park, PA, United States; ^2^ Oceana County Extension Office, Michigan State University, Hart, MI, United States; ^3^ Department of Entomology, Pennsylvania State University, University Park, PA, United States; ^4^ Department of Animal and Environmental Biology, University of Benin, Benin City, Nigeria; ^5^ Department of Entomology, University of Georgia, Tifton, GA, United States; ^6^ Penn State Extension, Pennsylvania State University, Leesport, PA, United States; ^7^ Department of Natural Resource Ecology and Management, University of Oklahoma, Stillwater, OK, United States; ^8^ Department of Biological Sciences, Pennsylvania State University, University Park, PA, United States

**Keywords:** red maple, black walnut, tree physiology, feeding damage, photosynthesis

## Abstract

Spotted lanternfly (SLF; *Lycorma delicatula* White; Hemiptera: Fulgoridae) invaded the US from Asia and was first detected in 2014; currently, populations have established in 14 states primarily in the Northeast and Mid-Atlantic. It feeds voraciously on phloem sap from a broad range of host plants, with a preference for tree of heaven (*Ailanthus altissima* [Sapindales: Simaroubaceae]), grapevines (*Vitis* spp. [Vitales: Vitaceae]), and several common hardwood tree species. We evaluated the impacts of fourth instars and adults confined to a single branch or whole trees on gas exchange attributes (carbon assimilation [photosynthetic rate], transpiration and stomatal conductance), selected nutrients, and diameter growth using young saplings of four host tree species planted in a common garden. In general, the effects of adults on trees were greater than nymphs, although there was variation depending on tree species, pest density, and time post-infestation. Nymphs on a single branch of red maple (*Acer rubrum* [Sapindales: Sapindaceae]), or silver maple (*Acer saccharinum* [Sapindales: Sapindaceae]) at three densities (0, 15, or 30) had no significant effects on gas exchange. In contrast, 40 adults confined to a single branch of red or silver maple rapidly suppressed gas exchange and reduced nitrogen concentration in leaves; soluble sugars in branch wood were reduced in the fall for silver maple and in the following spring for red maple. Fourth instars confined to whole silver maple trees reduced soluble sugars in leaves and branch wood, and reduced tree diameter growth by >50% during the next growing season. In contrast, fourth instars in whole tree enclosures had no effects on black walnut (*Juglans nigra* [Fagales: Juglandaceae]). SLF enclosed on tree of heaven at 80 adults per tree suppressed gas exchange after two weeks of feeding, but did not alter non-structural carbohydrates, nitrogen concentrations, or tree growth. Results suggest that moderate to heavy feeding by SLF on young maple saplings may impair tree growth, which could have implications for production nurseries and forest managers.

## Introduction

Plant responses to herbivory may include changes in both primary and secondary metabolism (1, 2). Chewing insects, such as caterpillars, are well known for causing extensive tissue damage and activating the jasmonic acid signaling pathway, leading to the production of a variety of plant defensive secondary metabolites (2), as well as having impacts on plant resource allocation (3). As damage from defoliation is more easily documented than the extent of feeding by piercing/sucking insects, impacts on plant primary metabolism by defoliators have received more attention than for sap-feeding herbivores, yet sap-feeders can also have pronounced effects on plant physiology (1, 4, 5). Sap-feeding herbivores consume carbohydrates and nutrients from phloem and/or xylem tissue, potentially reducing available energy and nutrients for above- and belowground growth of plants which can impact short- and long-term plant health. A meta-analysis conducted by Zvereva et al. (1) found that across studies from 52 papers, sap feeders usually reduced growth and photosynthesis, and that generalist herbivores had more negative impacts than specialists. However, changes in plant primary metabolism in response to sap-feeders can vary considerably among insect guilds and plant species (1, 3).

To our knowledge, other than the brown planthopper, *Nilaparvata lugens* Stål (Hemiptera: Delphacidae), no other planthoppers have been investigated for impacts on plant primary metabolism until recently (4, 5). This is likely because most planthoppers are not considered pests. *N. lugens* is a specialist on rice and a major economic pest throughout Asia (4). Feeding on susceptible rice plants reduces photosynthesis and interferes with translocation of nutrients, reducing plant growth (6, 7).

The invasive planthopper, spotted lanternfly (*Lycorma delicatula* White; Hemiptera: Fulgoridae; hereafter SLF), provides an opportunity to investigate the impacts of a generalist planthopper on host tree physiology. Unlike the specialist, *N. lugens*, SLF has a worldwide host range of 103 plant taxa, more than 56 of which have been reported as hosts in the US (8). It has a strong preference in its native and introduced ranges for tree-of-heaven (*Ailanthus altissima* [Sapindales: Simaroubaceae]) and wild and cultivated grape (e.g., *Vitis* spp.). Other frequent hosts in the US include several native deciduous trees including maples (*Acer* spp.), walnuts (*Juglans* spp.), birches (*Betula* spp.), and willows (*Salix* spp.). SLF is native to China and has spread to Vietnam, South Korea, Japan, and most recently the US (9). First detected in Berks County, Pennsylvania in 2014, SLF has established populations in 14 states in the US to date (10). SLF has proven to be a serious economic pest of several deciduous trees and agricultural crops, especially grapevines (11, 12).

SLF has four instars that develop from late spring to mid-summer before becoming adults in late July and early August (13). The adults feed on copious amounts of phloem sap to reach sexual maturity and begin laying eggs in late August or early September. Oviposition continues until the adults die from a hard freeze, usually in mid-November or early December. The long duration of the adult stage is particularly destructive to the health of cultivated grapes (11, 12). A recent study found that adult SLF feeding at increasing densities markedly reduced carbon assimilation (hereafter referred to as C assimilation) and late-season concentrations of non-structural carbohydrates and nitrogen, with the strongest impacts on belowground root carbon and nitrogen storage; reduced starch storage in the roots can lead to winter mortality of vines (5). C assimilation (photosynthetic rate) is the general term most widely used by plant ecophysiologists for fixing inorganic C (CO_2_) into organic C (carbohydrates) (14, 15).

While SLF likely co-evolved with the preferred hosts of grape and tree of heaven in its native range and can compete with grapevine sinks for resources leading to whole-plant carbon limitation (5), effects on the health and physiology of tree hosts native to the US have not been investigated. In the US, SLF frequently utilizes important ornamental and/or forest trees such as silver maple (*Acer saccharinum* [Sapindales: Sapindaceae]), red maple (*Acer rubrum* [Sapindales: Sapindaceae]), weeping willow (*Salix babylonica* [Malpighiales: Salicaceae]), black walnut, and river birch (*Betula nigra* [Fagales: Betulaceae]). In Pennsylvania alone, the annual economic losses from SLF for the ornamentals and forest industries are estimated at $8 million and $16.7 million per year, respectively (16).

Due to voracious SLF feeding and the tendency for them to aggregate in high numbers on individual trees (17), we hypothesized that SLF can modify the allocation of resources for defense against herbivory at the expense of growth in its hardwood tree hosts. Other than tree of heaven, healthy mature ornamental and forest trees have rarely been killed by SLF, although canopy dieback and plant health decline has been observed, with occasional mortality of saplings of black walnut (9) and maples (18). SLF also produce copious amounts of honeydew, which promotes the growth of sooty mold on plants below feeding sites, impeding photosynthesis of affected plants (19).

In 2019 and 2020, we measured C assimilation, transpiration, and stomatal conductance in response to SLF feeding pressure. We also evaluated concentrations of non-structural carbohydrates and nitrogen, as well as tree growth at increasing densities for silver maple, red maple, black walnut, and tree-of-heaven using planted saplings in a common garden. We used multiple methods to expose trees to SLF feeding. We began by confining SLF to single branches of young trees, followed by whole-tree enclosures, which more closely resemble field conditions. We hypothesized that plants, in response to feeding pressure, may initially compensate with enhanced gas exchange attributes, but that over time these variables would decline. We also hypothesized that the plants’ source/sink relationships would be altered as the insect competes for essential nutrients such as C and N. Besides competing directly with plant sinks, sap-feeding insects can change gene expression for the processes of N assimilation and translocation, such as in the brown planthopper on rice (4, 20) and SLF feeding on grapevines (21). We further hypothesized that tree growth would be reduced the following year.

It is important to note that in 2019 when we started this study, there was no published literature on the impacts of any fulgorid on its host plants to guide us, so in designing our experiments for which life stages to test on which host trees, we drew on our observations of the unusual tendency of frequent movement of this insect and what we know about factors that influence plant responses to herbivory. Based on 8 years of watching this fulgorid’s behavior in the wild and results from a nymph dispersal study we conducted in 2019 (22), we have observed that nymphs tend to move as often as every few days among different host plants but will arrest for a few weeks on black walnut as late-stage nymphs (Walsh and Hoover, pers. obs.), while they will stay on tree of heaven for long periods of time at any life stage (23). Despite SLF’s strong preference for tree of heaven (23–25), because its fitness is enhanced when it uses diet mixing (26), they tend to move off tree of heaven for periods of time to feed on other hosts and then move back (17) when they become late-stage adults. Adults then tend to move off trees of heaven when the trees begin to senesce, ending up on maples if they are available, or other hosts that have not yet entered dormancy (Walsh and Hoover, pers. obs).

## Materials and methods

To determine the impact of SLF feeding pressure on hardwood tree physiological responses, we used saplings for our study, which allowed us to confine SLF to the same tree for the duration of the experiments. We used two different types of field experiments over two years (2019 and 2020) in which fourth instars or adults were confined to: 1) a single branch in a sleeve cage or 2) whole-single-tree enclosures in a common garden in Berks County, PA. The methods used for measuring physiological plant responses and collecting tissue samples for total non-structural carbohydrate (TNC) and nutrient analyses were the same for both types of experiments. All insects used in the experiment were field collected. For gas exchange measurements in the whole-tree studies, we randomly selected a branch for each measurement date using young, fully expanded leaves each time. We did not use the same leaf at different time points because for some trees (tree of heaven and black walnut) the compound leaves made it difficult to use the same leaflet more than once, due to the compression applied to the leaf by the cuvette in the gas exchange instrument during the measurement process. For all experiments, the number of SLF that died each day (or nymphs that molted to adult in experiments with fourth instars) were recorded and replaced as needed for the duration of each experiment. Also, the number of replicates for each experiment was limited by the time required to take gas exchange measurements with the LI-COR instrument during the window of time solar radiation was equally available to all experimental and control trees (see detailed methods for measuring gas exchange below).

In planning the duration of our experiments, we considered several factors that influence tree primary metabolism in response to herbivory, including insect density and life stage, tree age and size, and the duration of feeding (14). We have found in the wild and in confined rearing of SLF that the combination of smaller and younger trees with the older insects produces greater effects on the plants (Walsh and Hoover, pers. obs). For example, in previous studies when rearing confined SLF on potted trees, we had to swap out tree of heaven, black walnut, and maples for fresh trees weekly or insect mortality was high and development was delayed. Also, as mentioned above, other than tree of heaven, most of the trees that are killed by SLF are saplings or trees of heaven (of any size) following heavy, prolonged feeding, especially by adults (18). Thus, due to the small size of the trees in this study, experiments were limited in duration by issues with increasing SLF mortality. Short-term studies of herbivores on primary plant metabolism are not uncommon in the literature in both herbaceous plants (27, 28) and forest trees (29).

### Experimental setup

In fall 2018, a 0.8-ha common garden was established in Blandon, PA consisting of four blocks of trees (hereafter referred to as Blocks 1-4). Each block contained an equal number of black walnuts, red maples, silver maples, and trees-of-heaven. Block 1 was planted in a completely randomized design with 25 trees per species for a total of 100 trees. These silver and red maples were planted from 26.5-L containers (2.5 and 3.5 years old, respectively), and black walnut (18 months old) from 7.6-L containers (Octoraro Native Plant Nursery, Kirkwood, PA). Tree-of-heaven plants were grown from seed in a greenhouse and transplanted when trees were 1.2-1.5 m tall. The larger trees in Block 1 were of sufficient size to use in the first year of this study (2019).

Blocks 2-4 were arranged in a randomized split-plot design and each plot contained an equal number of black walnuts, silver maples, red maples, and tree of heaven. Within each block there were four plots, one plot per tree species, and plots were randomized within each block. Within each plot there were 48 trees of the same species, and these plots were replicated in each of the 3 blocks. Thus, there were 144 trees per species for a total of 576 trees. Red and silver maple trees were transplanted from 2 to 3-year-old, 1-1.2 m tall, 3.8-L potted trees planted the year before from bareroot stock (Cold Springs Nursery, Doylestown, PA) and maintained in a greenhouse for one year before being transplanted. Black walnut trees (18 months old) were transplanted from 7.6-L pots (Octoraro Native Plant Nursery), and tree of heaven were transplanted from 1.2-1.5 tall, 3.8-L potted plants grown from seed in a greenhouse for one year. All trees were spaced 3 m apart and each block was spaced 3.5 m apart. A single row of buffer trees was planted to surround the entire garden using the same tree species as its neighbor to minimize edge effects. Drip irrigation and deer fencing were installed, while weed management, rodent control and winter pruning were performed by a commercial landscaper. Trees were fertilized in the fall of each year by Bosold Landscaping.

For each experiment we aimed to select trees of different species and within species that were similar in size, especially in caliper measurements (diameter at breast height, DBH). We avoided using the same trees for different experiments within the same year. Details for each experiment are shown in **Supplemental Table S1**.

### Impacts of SLF confined to a single branch using sleeve cages

To determine the impact of SLF feeding pressure on a single branch, two experiments were conducted at the common garden in 2019, one with fourth instar nymphs in July and one with adults in August using varying densities. The larger sizes of fourth instars and adults, as well as the damage incurred to vineyards by the influx of SLF late in the season, strongly suggest that these are the most impactful life stages (11). On July 18, 2019, 18 silver maple and 18 red maple trees were selected in Block 1 and randomly assigned to one of three treatments: control (0 insects), light density (15 nymphs/sleeve), or moderate density (30 nymphs/sleeve). Mean DBH of the maples in this block was 26.2 ± 0.69 mm (SEM) for red and 30.0 ± 0.84 mm for silver. There were 6 replicates per treatment combination (2 tree species x 3 density treatments) for a total of 36 experimental trees. Also, in this first year of experiments at the common garden in 2019, the only trees that were established and growing well enough to use were the red and silver maples in Block 1.

Sleeve cages were sewn from mesh fabric (Joann Fabrics, State College, PA) (68 cm x 28 cm) in the shape of a bag and closed at the proximal end of the branch with a cable tie to contain nymphs on healthy branches of experimental trees. A branch with no sleeve cage was flagged on each tree (controls and treatments) to determine if the sleeve alone impacted plant responses. Branches were approximately 0.8 cm in diameter. Fourth instars were field collected and introduced into the sleeves on July 21, 2019. Sleeves were checked for mortality and dead nymphs were replaced daily to maintain treatment densities. For each tree, one leaf from the branch in each sleeve cage and the flagged branch with no sleeve cage were used to measure gas exchange just before the introduction of nymphs (July 18) and again on the day the experiment was terminated (August 2) when nymphs were all molting to adults overnight and mortality was increasing (12-day experiment). Samples for carbohydrate concentrations were not taken for branches exposed to nymphs in sleeve cages due to no detectable effects of SLF on gas exchange (see **Suppl. Table 3B** results).

To determine if adult feeding pressure on a single branch impacts tree physiology, 20 red and 20 silver maple trees were randomly selected in Block 1 and assigned to one of two SLF densities, 0 or 40 adults/branch. Given that we had seen no effects of nymph feeding pressure on gas exchange measurements, we used a single high density of SLF to determine if we would see any effects of SLF on maples. We set up 10 replicates per treatment combination (10 replicates x 2 tree species x 2 treatment densities) for a total of 40 experimental trees. Adults were collected on September 24, 2019 and introduced into a sleeve cage on each experimental tree, while empty sleeve cages were placed on control trees. This study was terminated on October 4, 2019 when decline in photosynthetic rates of controls indicated trees were entering dormancy, confounding the variables we were measuring (11-day experiment).

Gas exchange measurements were made as described below on September 24 and 27 and October 1 and 4 using one leaf from the branch in each sleeve cage and one leaf from a flagged branch on each tree without a sleeve cage to determine if the cage alone affected results. For non-structural carbohydrate assays, branch samples were collected on the same dates as gas exchange attributes were measured. Tree roots were collected the following spring as soon as the ground thawed (February 11, 2020) and processed as described below.

### Impacts of SLF on trees using whole-tree enclosures

During the second year of this study when trees were more established, we investigated the effect of fourth instar and adult feeding pressure on plant physiology using whole-tree enclosures. This permitted insects to move around on the tree as they do in the wild. Specifically, it allowed them to freely select feeding locations, regulate body temperature, and minimize weather exposure, which helped to reduce insect mortality (see Results section below for details).

Enclosures for whole trees were constructed from the same mesh as the sleeve cages by sewing together a four-sided tube approximately 132 cm wide x 3.5 m tall with two sleeve openings on opposite sides that were closed with a cable tie for easy access to the foliage inside the cages. Enclosures were attached at the top of the trunk with a cable tie to a 3.3-m-tall steel pipe sunk into the ground 30-40 cm deep and distanced 60 cm from the trunk (to avoid damaging the roots). This was necessary to prevent the enclosures from falling over during windy periods. The enclosure covered the entire tree and was attached with a cable tie to the trunk just below the lowest branches to prevent escapes.

Black walnut trees in Block 1 and silver maple trees in Block 2 were randomly selected to be used for fourth instars at densities of 0, 40, 80, or 120 per tree. While fourth instars will feed on silver maple readily (20), they can complete their entire life cycle on mature black walnut (26); SLF are often observed in large numbers on single branches of black walnut as third and fourth instars, moving to maples when walnuts begin to senesce (15). Trunk DBH was 18.6 ± 0.55 mm for black walnut and 14.4 ± 1.20 mm for silver maple. There were five replicates per treatment combination (2 species x 4 densities x 5 replications) for a total of 40 experimental trees. On July 22, 2020, fourth instar nymphs were introduced into the enclosures. Leaf gas exchange was measured as described below prior to insect introduction (July 22) and on three additional dates following insect introduction (July 26, 29 and 31). Enclosures were checked daily for fourth instars that had died or molted overnight to adults and replaced with new fourth instars. After 10 days, the experiment was terminated when all fourth instars were molting to adult overnight. To quantify non-structural carbohydrates, branch and leaf stem samples were collected on July 22 and 31, 2020.

For the adult study, feeding pressure was evaluated on silver maple and tree of heaven that were randomly selected and assigned to treatments in Block 1; we used these larger trees to ensure adequate food for adults. On August 6, 2020, adults were introduced into whole-tree enclosures at densities of 0, 40, 80, and 120 per tree with 5 replicates per treatment for each tree species for a total of 40 trees. Tree diameters were 35.05 ± 1.39 mm for silver maple and 30.0 ± 1.33 mm for tree of heaven. Leaf gas exchange was measured as described below prior to insect introduction on August 6 and August 9, 12, 17, 20 and 25 (20-day experiment). To quantify non-structural carbohydrates and nutrients, branch and leaf stem samples were collected on August 6 and 25 and the experiment was terminated on August 25, 2020. Roots were sampled on April 7, 2021 to determine effects of prior year SLF feeding on belowground carbon stores after overwintering.

### Tree gas exchange measurements.

Attributes of gas exchange (C assimilation, transpiration, and stomatal conductance) were measured on one leaf per treated branch and for controls on the dates described above from 09:00 to 13:00 under saturating light conditions (1500 µmol m^-2^ s^-1^) using a LI-6400 Portable Photosynthesis System (LI-COR Biosciences; NE, US).

### Non-structural carbohydrate and nutrient analyses

Stem and root samples were taken from trees infested with SLF as described above. Samples were bagged, immediately frozen in dry ice, and transported to the laboratory for storage at -80°C for later processing. Samples were lyophilized at -50° C and 0.01 mbar negative pressure, then ground using a Wiley model 1 mill with a 2-mm-mesh screen. TNC concentrations were extracted from samples using a modified Somogyi-Nelson reducing sugar determination method (30, 31). This method uses enzymatic digestion to rapidly quantify reducing sugars (e.g., glucose) and starch concentrations and is a cost-effective approach to analyze multiple samples. Briefly, two, 5 mg subsamples of freeze-dried tissue from each shoot, leaf, and root sample were weighed into 2-mL microcentrifuge tubes for soluble sugar extraction and starch digestion. One mL of deionized water was added slowly to each tube to wet the sample material and tubes were boiled in a hot water bath for 20 minutes. Tubes were then removed from the hot water bath and immediately placed in an ice bath until cool. For the soluble sugar extraction, 100 µL of 0.5 M sodium acetate was added to each subsample. For starch digestion, 100 µL of 0.5 M sodium acetate containing 5 units of amyloglucosidase (E.C.3.2.1.3.) and 2.5 units of α-amylase (E.C.3.2.1.1.) was added to each subsample. Tubes were incubated for 24 h at 30°C. Digestion was stopped by boiling samples for 5 min. Tubes were immediately placed in an ice bath until cool.

For the colorimetric analysis, 500 µL of Nelson’s reagent A was added to soluble sugar and starch extracts in 5-mL plastic tubes. Extracts were diluted as necessary to ensure that sugar samples were within the range of the standard curve (0-120 µg glucose mL^-1^). Tubes were boiled for 10 min and immediately placed in an ice bath to cool. Once cool, 500 µL of Nelson’s reagent B was added to each tube followed by 3.5 mL of water. Samples were vortexed and placed in the dark for 30 min. Sample absorbance was read at 520 nm using a UV-1600PC Spectrophotometer (VWR, Radnor, PA). The difference in absorbance between the soluble sugar and starch determination tubes was used to calculate starch concentration. Note that this method does not detect sucrose because it is not a reducing sugar.

Stem, leaf, and root samples were submitted to The Pennsylvania State University Agricultural Analytical Services Laboratory for quantification of N by combustion (32) for some but not all experiments. We did not send samples for N combustion for experiments where no changes in gas exchange attributes or carbohydrates concentrations were detected in response to SLF feeding pressure.

### Statistical analyses

Statistical analyses were carried out using JMP Pro 16.1 (SAS Institute Inc., Cary, NC, USA). Linear mixed models were used to determine if SLF treatments had effects on various aspects of tree physiology (gas exchange, tissue carbohydrate and nitrogen concentrations, and tree growth) and daily SLF mortality. Given the non-normal distribution of response variables, which were evaluated with a Shapiro-Wilk W test, data were natural log-transformed ([ln X] +1) before mixed model analysis; the explanatory variables were not transformed. In cases when the log transformation was not sufficient to achieve the normality of distribution due to considerable skewness of the response variable, we used generalized linear mixed models (GLMM) with a gamma distribution and a log link function on untransformed data, which do not require normal distributions. The underlying assumptions of the models were tested, including the distribution and homoscedasticity of residuals, as well as homogeneity of variance across the SLF treatment categories (Leven’s test).

A range of explanatory variables was considered in mixed models as fixed effects, including the SLF treatment type (expressed as different SLF densities per tree enclosure), tree species, number of days since SLF were first released on the plant, and the interaction between them. Tree number was modeled as a random effect to evaluate the degree to which between-subject variability of individual trees influenced experimental outcomes. A forward selection procedure was used for choosing a model that best fit the data by adding explanatory variables to a base model one at a time. Overall model fit and parsimony were assessed based on *p*-values, the normality of distribution of model residuals, Akaike’s Information Criteria (AIC), and Bayesian Information Criterion (BIC). Statistical significance of differences between groups of categorical variables were evaluated with Tukey’s Honest Significant Difference (HSD) test on least square means, but for datasets with unequal variances we used the Games-Howell *post-hoc* test. The Games-Howell *post-hoc* test is used to compare all possible combinations of group differences when the assumption of homogeneity of variances is violated (33). This *post hoc* test is based on Welch’s degrees of freedom correction; it uses Tukey’s studentized range distribution for computing the p-values and compares the difference between each pair of means with appropriate adjustment for the multiple testing.

We used *p <* 0.05 to designate statistically significant differences between treatments, and *p <* 0.1 to designate marginally significant differences. This is an approach that has been used by others when sample size is limited in field experiments (5, 34), including for a study of impacts of SLF feeding on grapevines (5). It also avoids committing a Type II error (failure to detect a difference when there is one). Exact *p-*values are reported to facilitate data interpretation and transparency.

## Results

### Gas exchange in sleeve cages.

In 2019, fourth instar nymphs confined in sleeve cages at high density (30 SLF), while not significantly different from the controls, marginally enhanced photosynthetic rates of both silver and red maple leaves, while the low density (15 SLF) marginally suppressed rates at the end of the 12-day experiment (August 2, 2019) (**Supplemental Table S2**, **Figure S1**). Feeding of nymphs had no effects on stomatal conductance or transpiration.

**Figure 1:**
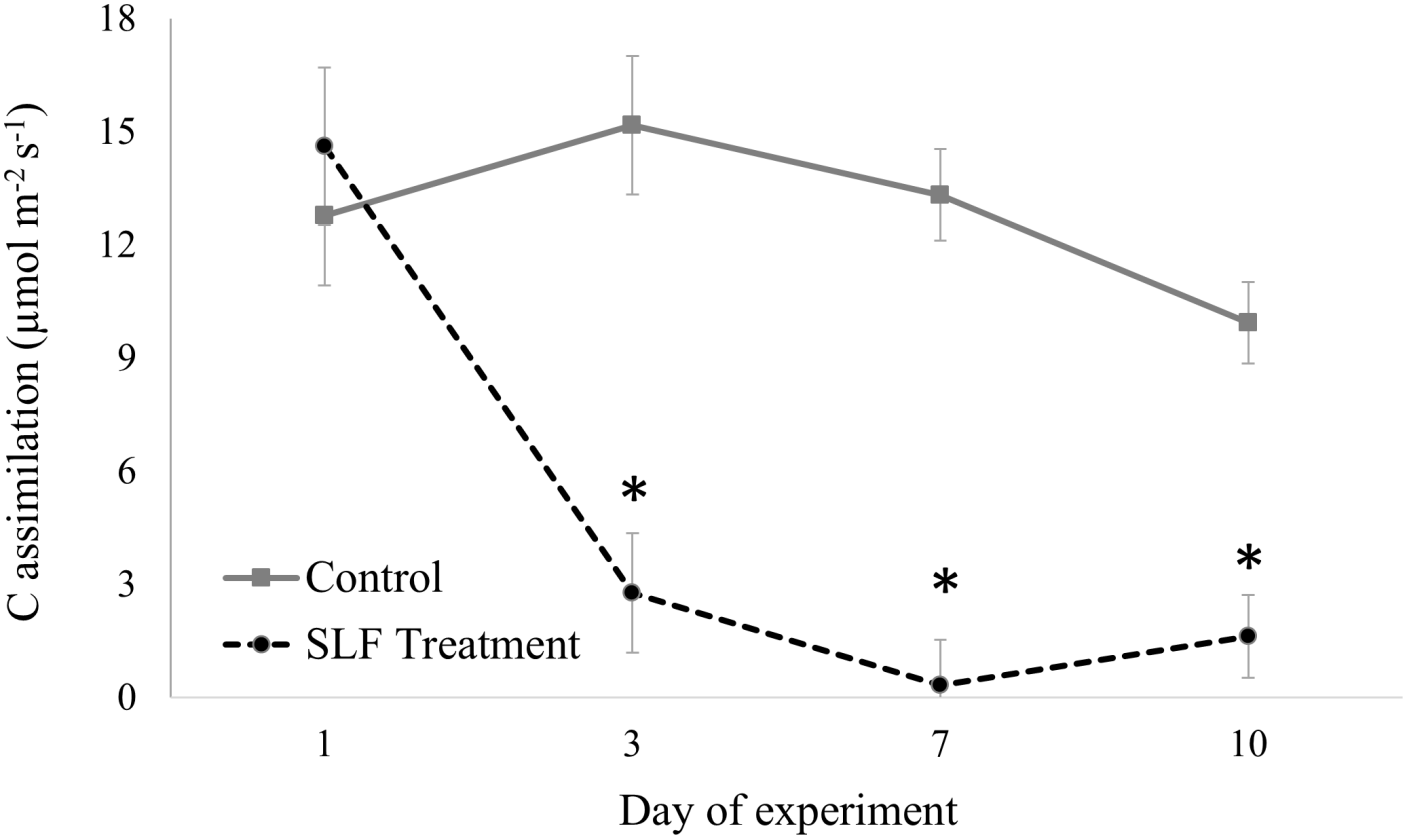
C assimilation over time measured on leaves in sleeve cages at the common garden in 2019. SLF adult densities were 0 or 40 per sleeve cage. Measurements were made on Sept. 24 and 27, and Oct. 1 and 4, 2019. Asterisks denote dates when rates on treated trees were significantly different from controls (*p* < 0.05).

In contrast to nymphs, sleeve cages containing 40 adults confined to a single branch significantly suppressed C assimilation by 4- to 20-fold compared to controls by day 3 (*p*<0.0001, **Figure 1**). The full mixed model with measurement date, tree species, SLF treatment type (branch with or without SLF) and interaction terms of these explanatory variables showed that the degree of influence differed by day of the experiment (**Table S3A**). C assimilation for all trees started at about the same rate at the beginning of the experiment, but at day 3 (*p* = 0.0252), 7 (*p* = 0.0050), and 10 (*p* = 0.0349), SLF feeding significantly suppressed C assimilation compared to controls. Sleeve cages alone had no significant effect, as evidenced by comparing C assimilation measured on branches with sleeves and no SLF to those without sleeves on both treatment and control trees (**Supplemental Table S3B**).

**Figure 2:**
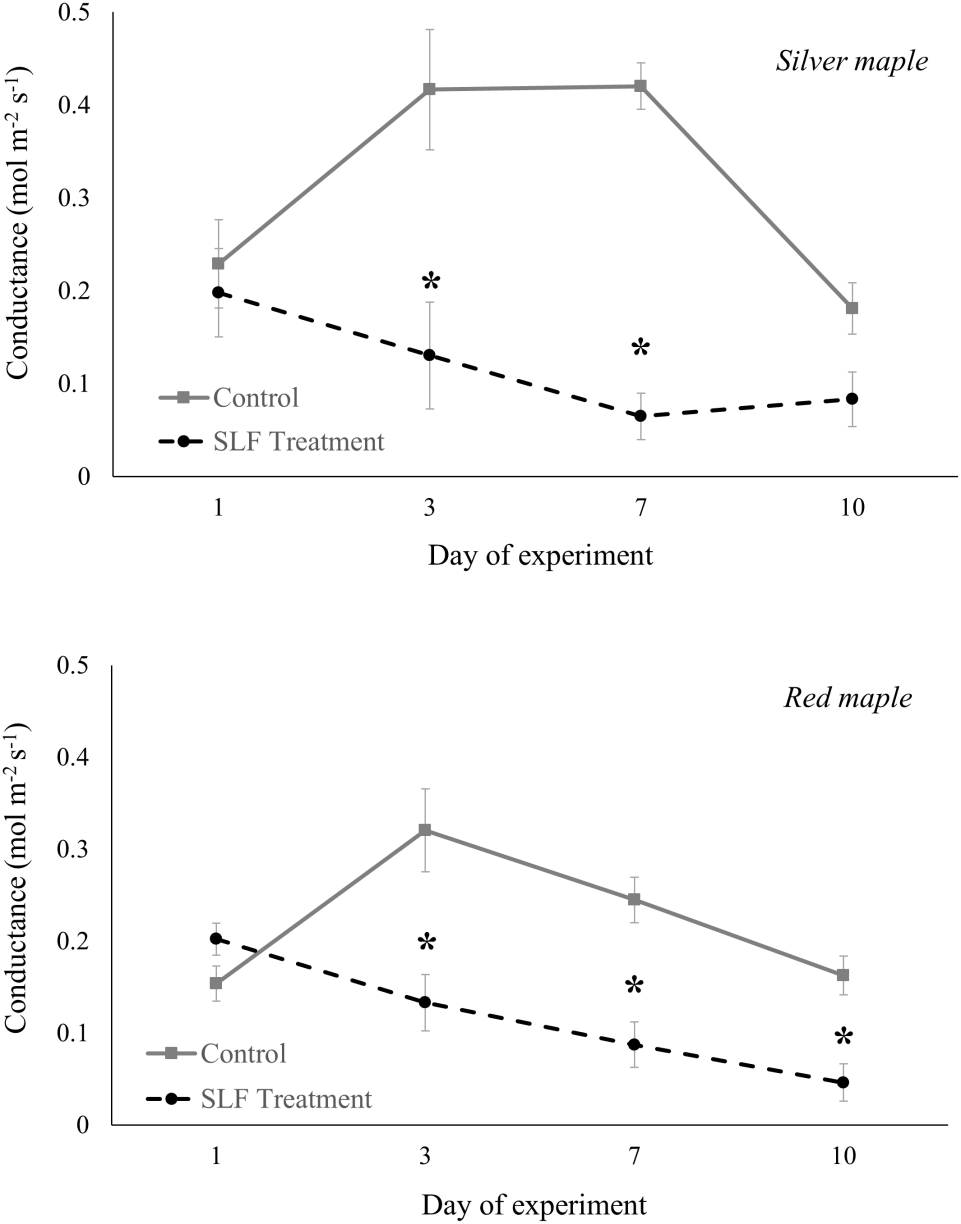
Stomatal conductance of silver maple and red maple by SLF treatment measured on leaves in sleeve cages at the common garden in 2019 on Sept. 24 and 27, and Oct. 1 and 4. Spotted lanternfly adult densities were 0 or 40 per sleeve cage. Asterisks above the standard error bars denote dates when conductance on treatment trees were significantly different from controls (*p* < 0.05).

**Figure 3:**
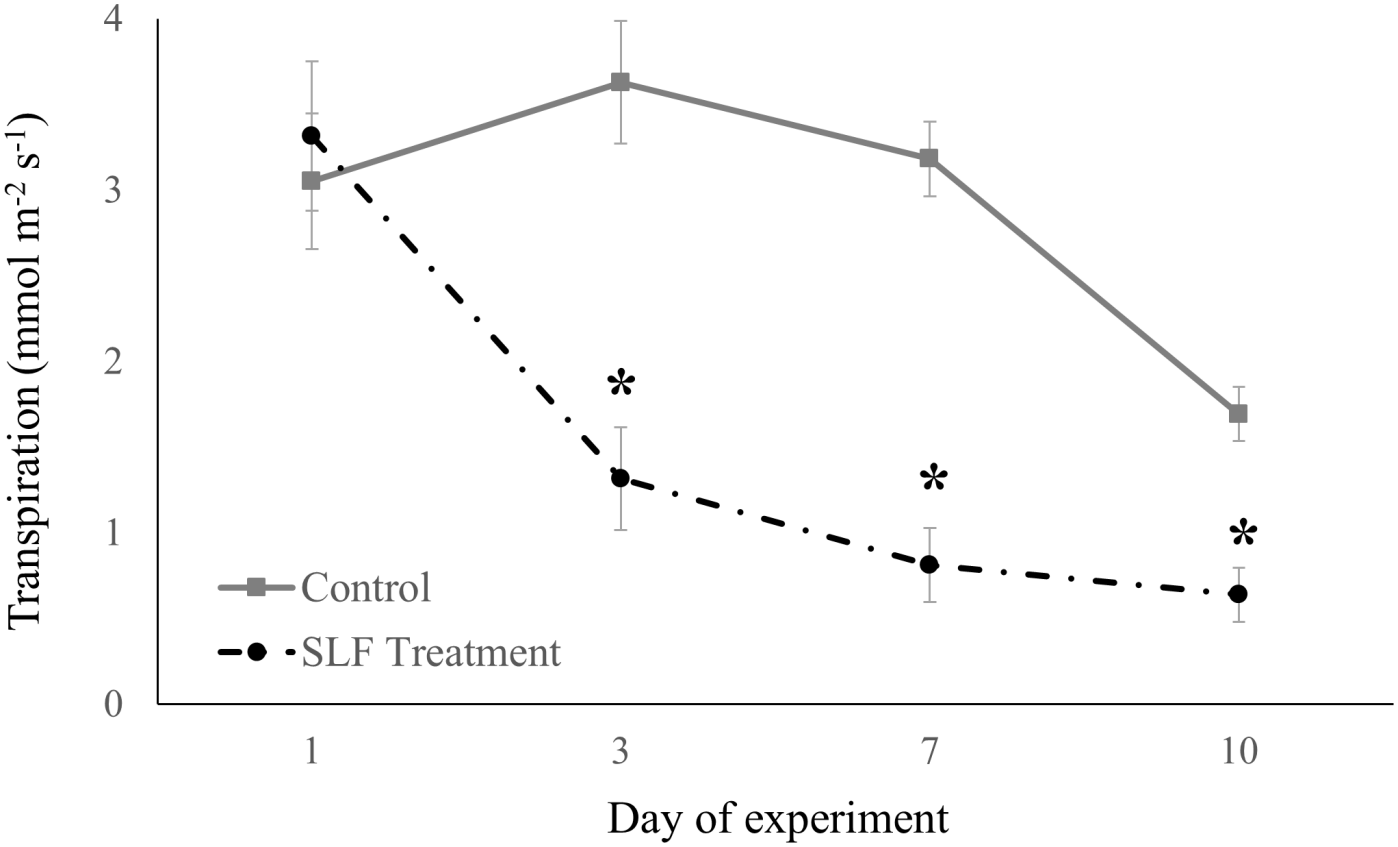
Transpiration measured in sleeve cages at the common garden in 2019 on Sept. 24 and 27, and Oct. 1 and 4 in response to 40 SLF adults per cage compared to controls. Asterisks above the standard error bars denote dates when transpiration rates on treatment trees were significantly different from controls (*p* < 0.1).

Adult feeding also altered stomatal conductance and transpiration. Mean conductance decreased by 51% for red maple (*p* = 0.0007) and 65% for silver maple (*p*<0.0001) on branches fed on by SLF. Feeding also affected the temporal patterns of conductance in a similar manner for both tree species (**Figure 2**); trees with no SLF infestation showed peak conductance in the middle of the experiment, whereas for infested trees, conductance steadily declined. The response of transpiration (**Figure 3**) to feeding did not differ between tree species, but rather depended on the day of the experiment (*p*<0.0001), declining sharply on infested branches, increasing on control tree branches by Day 3, then gradually declining on all branches towards the end of experiment (mean decline was 47% for the whole experiment).

### Gas exchange in whole-tree enclosures

In 2020 using whole-tree enclosures, we documented effects of feeding by fourth instar nymphs on gas exchange attributes; in general, these metrics were higher for black walnut than silver maple across SLF densities. C assimilation tended to increase slightly over time for all treatments (40, 80, and 120 per tree) for black walnut compared to controls and tended to decrease slightly over time for silver maple except for the last date (**Supplemental Figure S2**); however, these effects were not statistically significant for the 10-day duration of the experiment (**Supplemental Table S4A**). There were also no significant effects on stomatal conductance or transpiration at any nymph densities.

**Figure 4:**
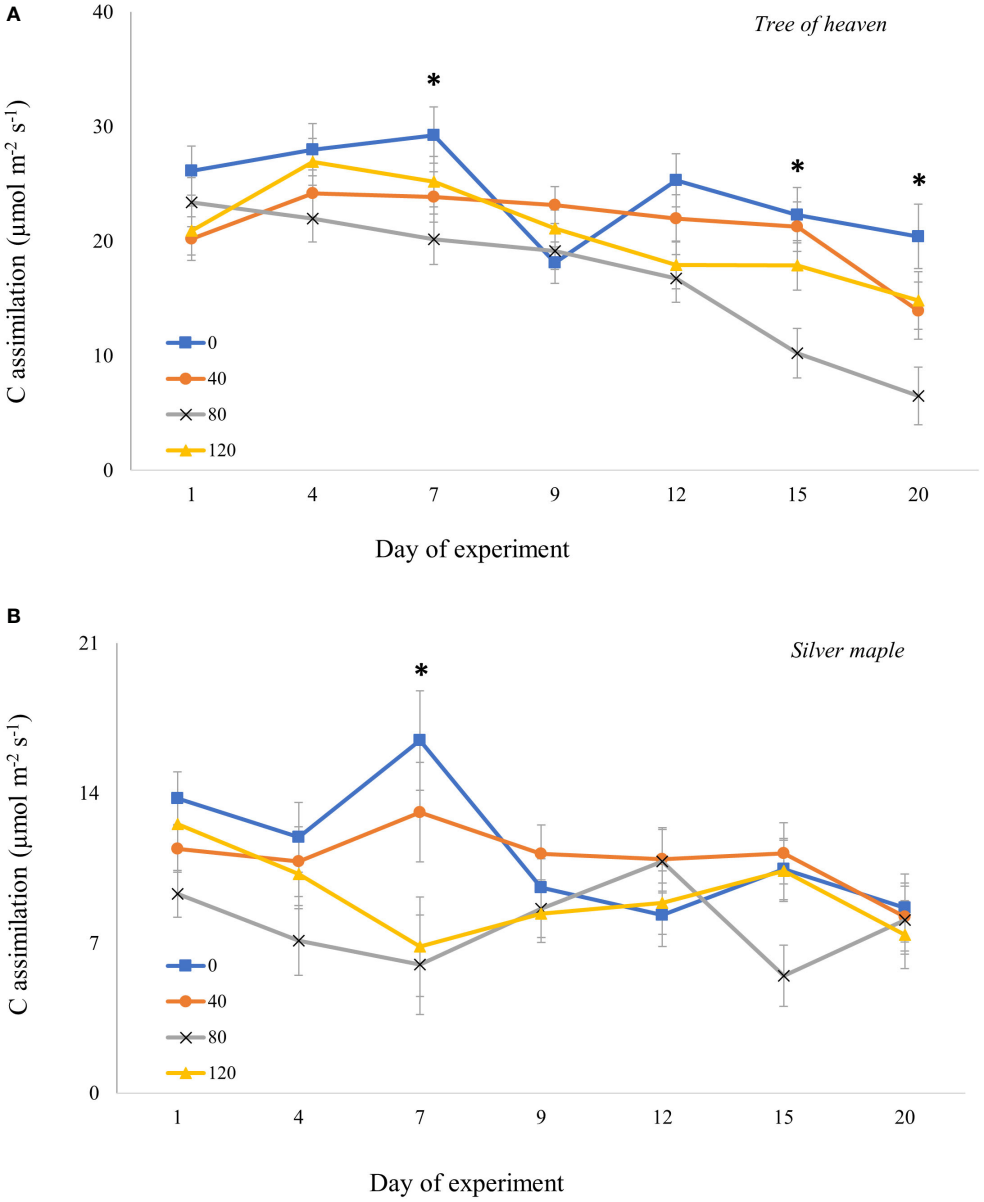
Carbon assimilation (photosynthetic rate) over time for tree of heaven **(A)** and silver maple **(B)** at the common garden. Spotted lanternfly adult densities were 0, 40, 80, and 120 per whole-tree enclosure. Note the differences in photosynthesis rates on the y-axes. Asterisks above the standard error bars denote dates when C assimilation rates on treatment trees were significantly different from controls (p < 0.05).

For adults confined to whole trees for 20 days, the overall model revealed that responses in gas exchange to different densities of adult feeding pressure were dependent on tree species and time post-infestation (**Supplemental Table S4B**). For both tree species, moderate (80 adults/tree) and high-density (120 adults/tree) feeding pressure significantly suppressed C assimilation (*p* = 0.0001 and *p* = 0.0433, respectively) compared to controls. However, the timing of the responses differed between tree species. For tree of heaven (**Figure 4A**), C assimilation by trees exposed to adults with moderate density was marginally lower than for controls on Day 7 (*p* = 0.0680) and declined on Day 9 for all trees including controls exposed to adults. On later dates, however, C assimilation for control trees recovered to near previous levels then gradually decreased, while there was no recovery for treated trees; instead, C assimilation continued to decline sharply. On day 15, C assimilation was significantly suppressed at moderate adult SLF density by 54% compared to controls (*p* = 0.0349) and by 52% compared to low SLF density (*p* = 0.0356). On the last day of the experiment (Day 20), C assimilation for moderate SLF density was significantly lower than controls (*p* = 0.0320), but not significantly different from the low-density treatment (*p* = 0.1669).

For silver maple (**Figure 4B**), at 7 days post-infestation, the moderate density of SLF marginally suppressed C assimilation by 65% compared to control trees (*p* = 0.0509, Tukey *post-hoc* test). These differences were not significant for the remainder of the experiment.

Temporal patterns and the magnitude of changes in conductance and transpiration responses to adult SLF feeding largely mimicked those of C assimilation, gradually declining with increasing pest pressure (**Supplemental Figures S3**, **4**; **Supplemental Table S4B**). Transpiration and stomatal conductance for tree of heaven declined significantly for trees with low SLF density (*p* = 0.0062 and *p* = 0.0234, respectively) compared to control trees on Day 4 (**Supplemental Table S4B**). On the last measurement date, transpiration and conductance for control trees or trees with low and high density SLF were not different from each other, but the trees with moderate densities of SLF had lower levels than the controls (significantly lower on day 20 for transpiration *p* = 0.0031 and conductance *p* = 0.0053). Transpiration and conductance in silver maple declined for trees with moderate and high-density adult infestations, but trees with light infestation had similar transpiration rates to the control. The difference between moderate density and controls were marginally significant on Day 4 (transpiration lower than controls by 48% and conductance by 42%). However, on Day 7 transpiration was significantly reduced by 67% and conductance by 63%. For high density, these variables were significant only on Day 7 (transpiration lower by 64%, conductance by 63%).

### Non-structural carbohydrates in sleeve cages

**Figure 5:**
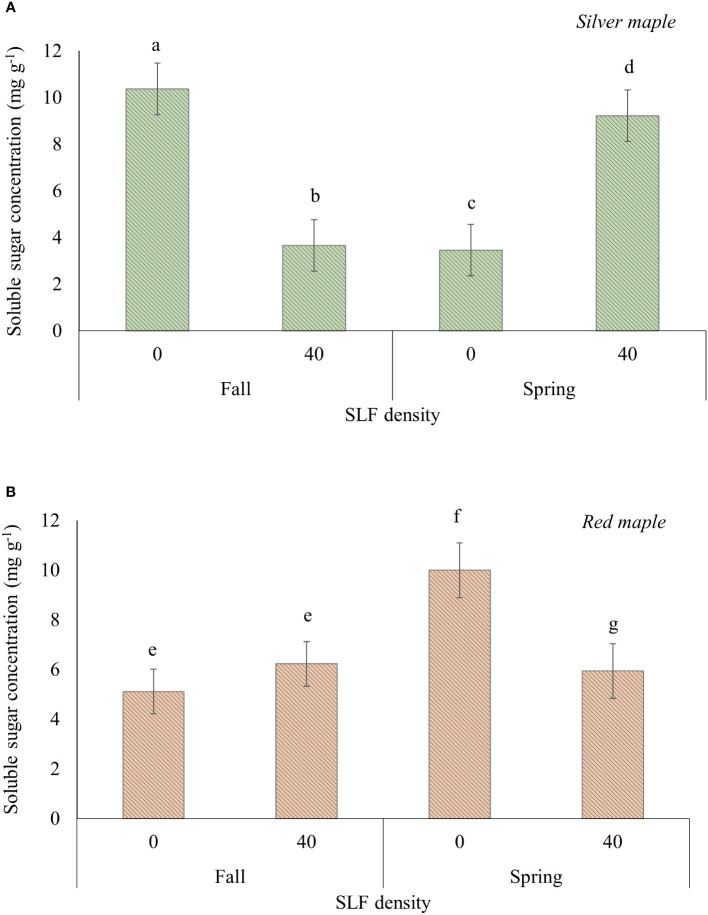
Average (least square means) concentrations of soluble sugars in branch woody tissue of silver **(A)** and red maple **(B)** trees exposed to SLF adults in sleeve cages. Branches were collected on October 4, 2019 (fall sampling), and March 27, 2020 (spring sampling). Carbohydrate concentrations are given in mg of glucose equivalents per g of dry tissue. Significant differences (*p* < 0.05) in soluble sugar concentrations between treatments for a given tree species within a sampling season are shown as different letters above the standard error bars.

SLF feeding significantly reduced the concentrations of soluble sugars in wood tissue of branches (mostly xylem), and this response differed by tree species and season of when samples were taken (**Table S5A**). For wood tissues in branches sampled in the fall immediately after the end of the experiment, soluble sugar concentration was reduced by 65% in silver maple branches treated with SLF compared to controls (*p* = 0.0059, Tukey test), but not in red maples (**Figure 5A**). However, there were no significant differences in TNC, soluble sugars (glucose equivalents), or starch concentrations in roots or bark of silver or red maple trees resulting from exposure to adult SLF in sleeve cages in the 2019 experiment (**Supplemental Tables S5A, B**).

Branches fed on by SLF and sampled again the following spring (2020) after overwintering had soluble sugar concentrations in woody tissue that were 62% higher in silver maple (*p*<0.0001) and 40% lower in red maple (*p* = 0.043), compared to controls (**Figure 5B**).

### Non-structural carbohydrates in whole-tree enclosures 2020

**Figure 6:**
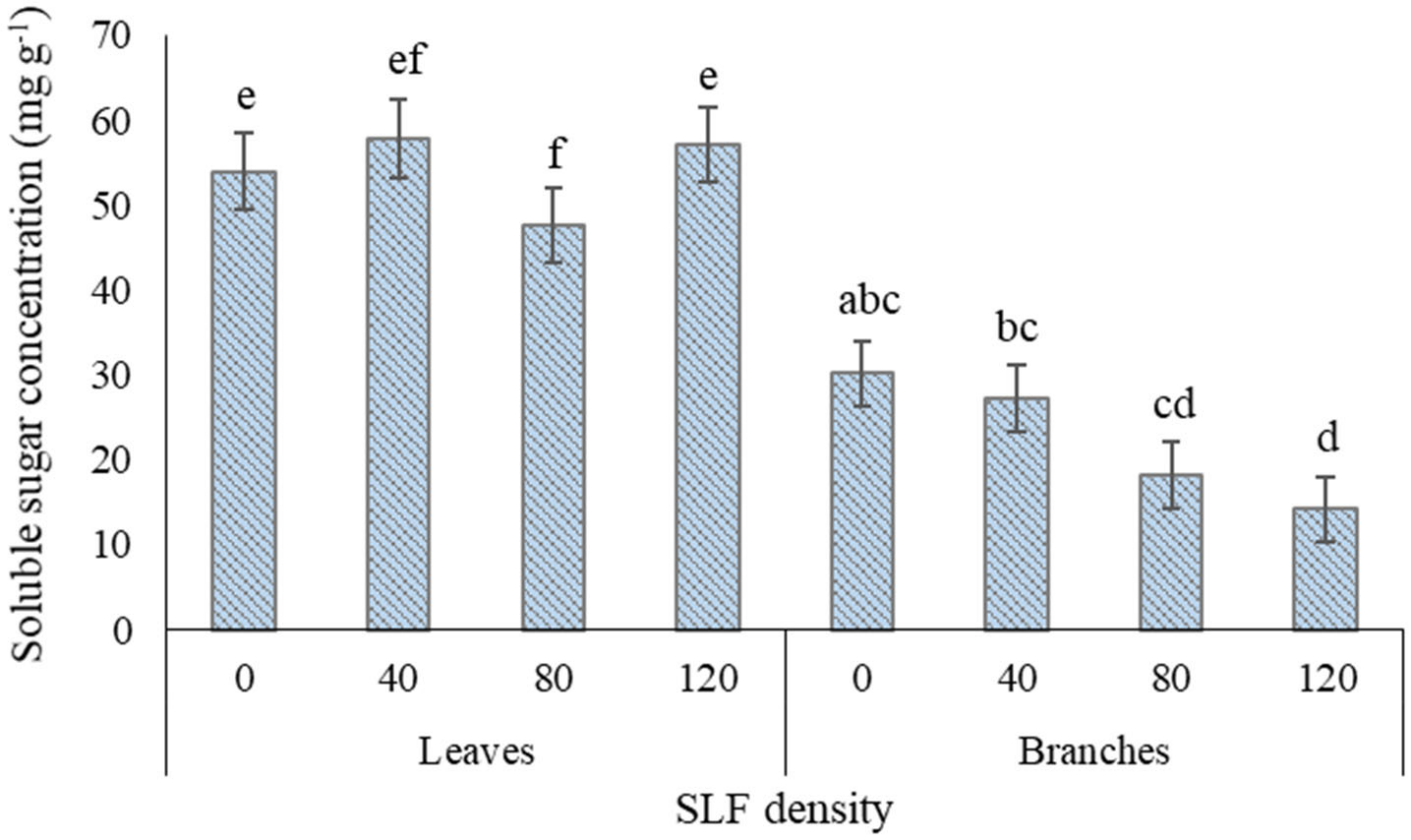
Average (least square means) concentrations of soluble sugars in leaf and branch tissue of silver maple exposed to varying densities of fourth instar SLF nymphs during the whole-tree enclosures experiment. Leaves were collected on July 22 and 31, 2020, and branches were collected on July 31, 2020. Carbohydrate concentrations are given in mg of glucose equivalents per gram of dry tissue weight. Significant differences (*p* < 0.05) in fraction of soluble sugars for leaves and in soluble sugar concentrations for branches between treatments within a tissue type are shown as different letters above the standard error bars; standard errors are for each mean total non-structural carbohydrate concentration.

SLF treatment with the moderate density of fourth instars (80 per tree) significantly reduced the fraction of soluble sugars to TNC by 23-29% in leaves of silver maples compared to control trees (*p* = 0.0433, Games-Howell *post-hoc* test) and compared to high SLF density (*p* = 0.0170) (**Supplemental Tables S6A, B**, **Figure 6**). Carbohydrate concentrations in branch woody tissue for silver maple declined in a density-dependent manner in response to nymphs; soluble sugar concentrations were 53% lower on average in silver maples exposed to high-density nymph feeding pressure (*p* = 0.0029) compared to controls, and 33% lower compared to trees with low nymph density (*p* = 0.0138) (**Supplement Table S6B**, **Figure 6**). TNC concentrations also decreased significantly in silver maple branches by 40% in trees exposed to high SLF density compared to controls (*p* = 0.0211), and by 41% compared to low SLF density (*p* = 0.0185). Carbohydrate concentrations for black walnut were unaffected by 4^th^ instar feeding pressure at any density (**Supplement Table S6A**).

Adults in whole-tree enclosures had no significant effects on carbohydrates for any of the tissue types (roots, branch, leaves) collected from silver maple or tree of heaven at any density (**Supplement Table S7**).

### Effects of SLF feeding on nitrogen

Independent of tree species, leaves from trees with adult SLF confined to a branch in sleeve cages had 20% lower N concentrations compared to controls (*p* = 0.0205) and a 25% higher ratio of carbon to nitrogen (*p* = 0.0447) in leaves compared to trees without SLF (**Table S8**). We did not detect any significant effects of adults on nitrogen in roots or leaves collected from silver maple or tree of heaven in whole-tree enclosures at any SLF density (**Supplement Table S9**).

### Tree growth

**Figure 7:**
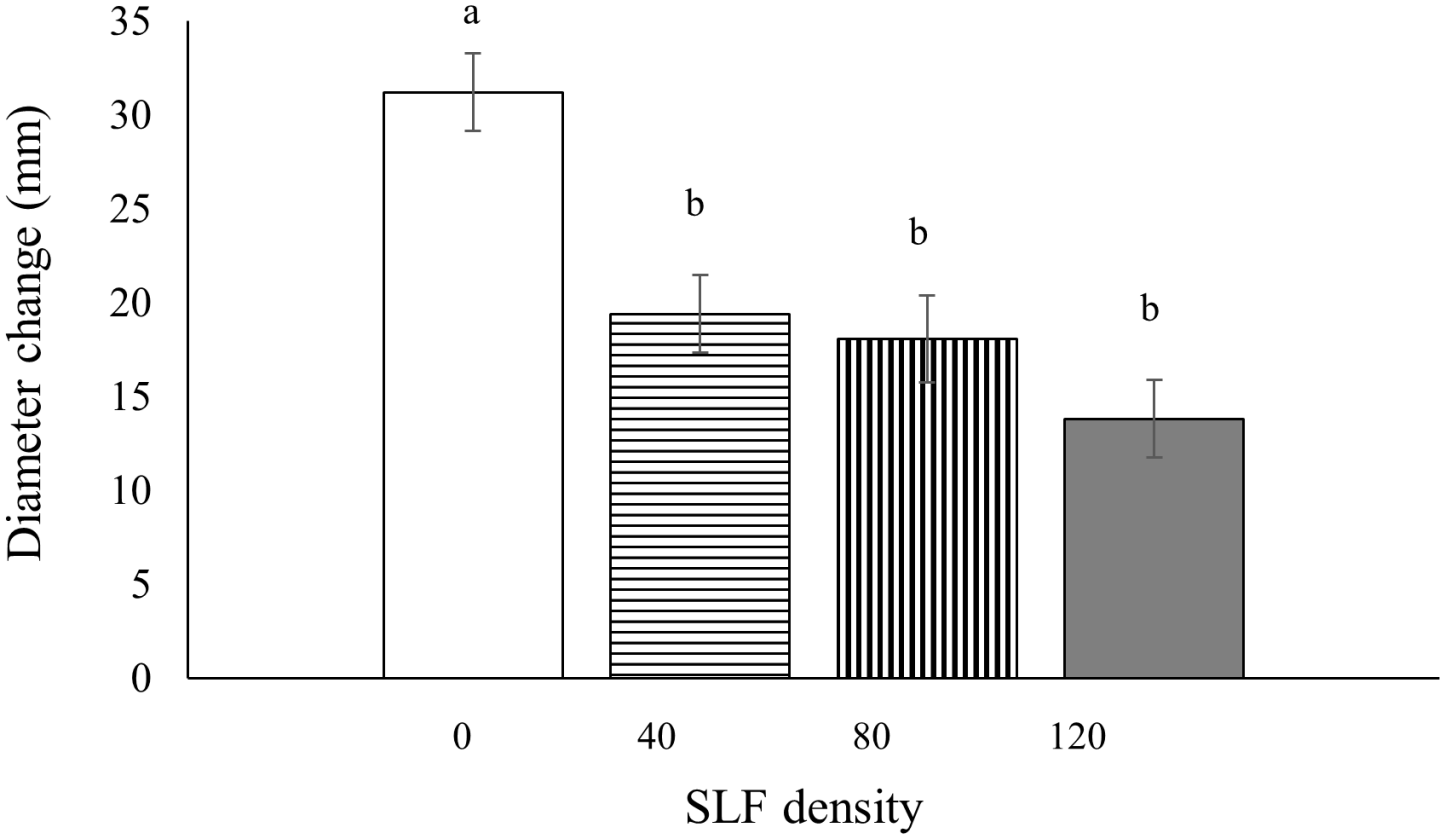
Mean tree diameter growth (at breast height) for silver maple during the growing season following the 2020 experiment as affected by SLF fourth instars feeding at different densities. Significant differences (*p* < 0.05) between treatments shown as different letters above the error bars. Tree diameters were measured on April 7, 2021 and March 15, 2022.

Tree diameters (at breast height, 1.4 m) were measured before and after the whole-tree enclosure experiments to determine if SLF feeding impacted tree growth; we did not document diameter growth of trees exposed to SLF in sleeve cages because we did not expect effects on whole-tree growth from feeding a short time on a single branch. Silver maples exposed to fourth instars in whole tree enclosures showed significantly stunted diameter growth during the next growing season (2021), and the decline was proportional to SLF density. Compared to controls, treatments with the highest SLF density reduced diameter growth by 55% (*p* = 0.0005), by 42% for moderate density (*p* = 0.0139), and by 38% for low density (*p* = 0.0359) (**Figure 7**). There were no significant effects from fourth instars on growth of black walnut trees in the same experiment. There were also no effects on diameter growth of silver maple or tree of heaven in response to feeding by SLF adults in whole-tree enclosures for three weeks during the same season (2020) or when measured the following growing season (2021).

### SLF mortality during experiments

For all experiments, enclosures were checked daily for mortality and dead insects were replaced to maintain constant feeding pressure by the same life stage present at the start of the experiment. In 2019, mortality of fourth instars in sleeve cages depended on tree species (*p* = 0.0355) and day of the experiment (*p*<0.0001); more nymphs died on red maple (mean of 18 ± 1.1% per day) than on silver maple (mean of 11 ± 1.1% per day), and the proportion of insects that died increased towards the end of experiment for both tree species. Tree number as a random effect was also significant (*p* = 0.0021), indicating high variability in percentage mortality of SLF among trees within treatment. There was no difference in the percentage of insects that died as a function of SLF density (15 or 30 fourth instars/sleeve).

Mortality of adults in sleeve cages in 2019 was greater than for nymphs and differed by tree species (*p*<0.0001) and day of the experiment (*p* < 0.0001). A higher percentage of SLF died in sleeves on red maple (43.6 ± 3.1% per day) than on silver maple (30.9 ± 3.1% per day), increasing towards the end of the experiment for both tree species. Tree number as a random effect was not significant (*p* = 0.2424).

**Figure 8:**
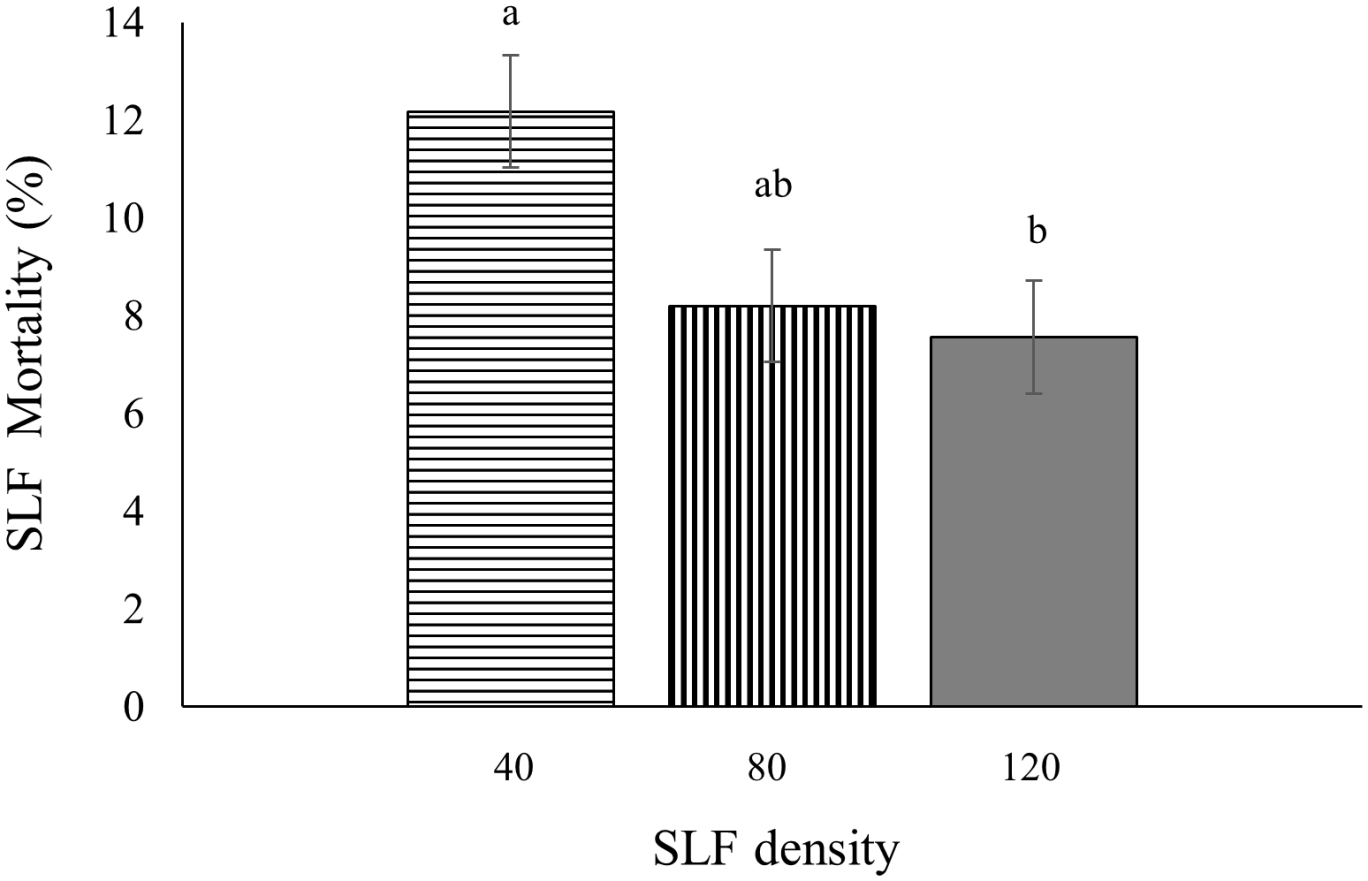
Mean percentage mortality per day for fourth instars in 2020 was influenced by SLF density in whole tree enclosures. Significant differences (*p* < 0.05) between treatments shown as different letters above the standard error bars.

In 2020, fourth instar mortality in whole-tree enclosures depended on tree species (*p* < 0.0001), day of the experiment (*p* =0.0005), and SLF density (*p* = 0.0156). There was a higher mean percentage of dead nymphs on silver maple (12.7 ± 0.64% per day) than on black walnut (5.9 ± 0.64% per day). Mean daily mortality decreased gradually until Day 6 for black walnut and Day 4 for silver maple, then went up towards the end of the experiment for both tree species. On Days 4 (*p* = 0.0333), 5 (*p* = 0.0029), and 6 (*p* = 0.0011) there were significantly lower percentages of daily mortality than on the first 3 days of the experiment. Interestingly, daily mortality decreased with increasing number of SLF per enclosure; at low density daily mortality was the highest (12%, *p* = 0.0227), and was significantly greater than in the enclosures with high SLF density (7%), while the moderate SLF density was intermediate (8%), which were not significantly different from the other treatments (**Figure 8**). Tree number as a random effect was significant (*p* = 0.0143).

Not surprisingly, adult mortality was markedly lower in whole-tree enclosures than in sleeve cages with daily mortality depending on tree species (*p* <0.0001) and day of the experiment (*p* <0.0001). Overall, adult SLF mortality on whole trees was generally low; mortality on silver maple was 5% per day and on tree of heaven it was 2% per day. For both tree species, mortality increased slightly until Day 4, followed by a decrease towards Day 8, then leveling off towards the end of experiment. Tree number as a random effect was significant (*p* = 0.0043), indicating high variability in percentage mortality of SLF among trees within treatment. There was a similar trend in mortality as a function of SLF density for adults as for nymphs, with higher mortality in enclosures with the lowest density, but these differences were not statistically significant (*p* = 0.2700).

## Discussion

Plants use a variety of strategies to tolerate and defend against herbivory; they may shift rates of photosynthesis and alter allocation of carbon and nitrogen resources to growth (3, 5), or to induced plant defenses (1, 19). Some plants respond by reducing carbohydrate reserves available for overwintering (5) and for growth the next year (26). In young grapevines, adult SLF feeding was shown to have dramatic effects on C assimilation and belowground starch reserves and these effects were density dependent (5).

We found that SLF adults confined to a single branch, in contrast to nymphs in sleeve cages, rapidly suppressed C assimilation, transpiration, and stomatal conductance for both red and silver maples, which was visible by 3 days post-infestation and continued for the duration of the experiment. Stomatal conductance was slightly more reduced for silver maple relative to red maple (65% vs. 51%, respectively, compared to controls), but it’s important to note that daily percentage insect mortality was also lower on branches of silver maple than on red maple. In addition, nymphs in sleeve cages suppressed nitrogen concentrations in leaves of both maple species, but this did not occur in response to adult feeding. These findings may explain why we rarely see heavy feeding by fourth instars on maples in the wild; they tend to move to maples in mid-September as adults, so it’s possible that the phenology of this host plant-insect interaction coincides with the timing during which SLF can more successfully tolerate tree defenses and/or obtain sufficient nutrients.

Soluble sugars in the wood of branches and nitrogen concentrations of both maple species in response to fourth instars declined markedly by the end of the experiment in the fall but recovered and was at higher concentrations for silver maples than controls the following spring, but not for red maple. This suggests that SLF can manipulate carbon allocation in these trees, but at different times points; soluble sugar concentrations in silver maple recovered by the next season but reduced soluble sugars in red maple were not evident until the trees had overwintered.

We expected fourth instars to reduce gas exchange attributes, TNC, and growth in black walnut trees given that this is a preferred host for late-stage nymphs (27) and that dieback is often observed when SLF congregate on mature black walnut branches in forests (28), parks and residential neighborhoods (Walsh and Hoover, pers. obs.). Instead, fourth instars had no effect on any variables we measured when given access to black walnut in whole-tree enclosures. In the field in July and early August, it is common to see overlapping life stages of SLF on trees and, thus, branches of black walnut may be heavily fed upon for several weeks as new third instars arrive and molt to fourth instars, prolonging the time late-stage nymphs feed on single black walnut branches. This pattern of movement is especially noticeable on mature black walnuts where fourth instars may also remain on branches after molting to adult until black walnuts begin to senesce. We also did not observe any wilting or branch dieback on these trees, so we suspect that our experiment on black walnut was too short (10 days) to reproduce the physiological impacts that can occur in the field with heavy late stage feeding pressure. However, because our aim was to examine the effects of fourth instars and not adults on black walnut, we terminated the experiment when every fourth instar was molting to adult overnight and mortality on this host was increasing, producing confounding factors.

While we did not find significant reductions in C assimilation by fourth instars in whole-tree enclosures in 2020 for silver maple, the soluble sugar (glucose equivalents) to TNC ratios were reduced in leaves in response to moderate feeding pressure compared to controls and compared to the other nymph densities. Also, soluble sugars in branch wood were reduced by half for silver maples fed on by the high density of nymphs compared to controls and by one-third compared to trees fed on by the low density of nymphs, indicating that SLF was able to manipulate carbohydrates in a density-dependent manner. Interestingly, the reductions in soluble sugars in leaves and branch wood of silver maples co-occurred with reductions in tree diameter growth in a density-dependent manner. Growth was reduced by more than half at the high SLF density, and reductions in growth gradually declined as SLF density decreased. This finding may have economic implications for maple saplings in production nurseries or regenerative growth in forests where slower growth in response to high SLF populations could be costly.

An unexpected finding was that the percentage mortality of nymphs in whole-tree enclosures was inversely related to SLF density; a greater percentage of nymphs died daily at the lowest SLF density than at higher densities for both silver maple and black walnut. It is possible that larger numbers of SLF were better able to manipulate resource allocation, as was shown for SLF adults on grapevines as the density of SLF increased (5). This may occur through a greater volume of salivary enzymes injected into the phloem during feeding at higher SLF densities, reducing the ability of the plant to limit sap flow by callose formation. This would be consistent with a previous report that the aphid *Megoura viciae* can prevent sieve tube plugging in the phloem using salivary proteins during feeding, which provides aphids with access to a continuous flow of phloem sap (29). Note that a similar trend in higher proportional mortality at lower SLF densities was also observed for adults on tree of heaven and silver maple, but these results were not statistically significant, perhaps due to the high variability among trees within treatment (tree number was a significant random effect for every experiment except for adults in sleeve cages).

The effects of adults when given access to the whole tree, and especially to the trunk where adults frequently feed, were more subtle than results of adults confined in sleeve cages. Suppression of gas exchange was greater and more consistent for tree of heaven than for silver maple, becoming evident after 2 weeks of feeding pressure. Adult feeding on whole trees also did not affect carbohydrate concentrations or tree diameter growth. For silver maple, we suspect that fourth instars may have had a stronger effect than adults due to differences in tree size, tree age, and feeding location. Silver maples exposed to fourth instars in our experiments were 2 years younger and less than half the diameter of the silver maples used for adults. Herbivores tend to have a greater impact on younger, smaller trees (14), and high SLF populations can kill saplings, whereas this rarely happens to larger trees, with the exception of tree of heaven that have been fed on heavily for several months, especially if they are attacked multiple years in a row (9, 15). In addition, since fourth instars tend to feed on branches rather than the trunk, their feeding activity is physically closer to where gas exchange, carbohydrates, and nitrogen were measured.

Our findings were similar, but not as consistent, as responses documented from heavy adult SLF feeding on young grapevines. In a recent study, intensive, continuous, late-season feeding by large adult SLF population densities (70 to 200 SLF per vine) significantly reduced gas exchange attributes in young grapevines (5). Adult SLF were found to compete with grapevine sinks for resources, leading to whole-plant carbon and nitrogen limitation, especially in the roots. In the US, the greatest economic impact from SLF introduction has been damage to vineyards. In Pennsylvania, it is not uncommon to find >100 SLF adults feeding on a single vine (12), and this heavy, repeated phloem-feeding can strongly reduce grape yields (up to 90%), fruit quality, and, in some instances, kill vines (11). In contrast, SLF rarely kill trees in the field other than occasional young saplings, and tree of heaven of any size, in response to long-term heavy feeding (18). In the field, tree of heaven is the preferred host for every life stage; thus, feeding pressure, especially by adults, can be very heavy for several months.

A potential explanation for failure to detect significant differences in plant physiology in all experiments may be the high degree of variability in our explanatory variables among trees within treatment. Tree number was a significant random effect in most experiments, except for adults confined to a single branch where impacts from SLF were overwhelming, suggesting that in most cases there was considerable variability from tree to tree within treatment, including in the ability of each tree to support the same number of SLF individuals. This lends credence to the term “hot” tree used by SLF researchers to describe high densities of SLF (nymphs or adults) on one tree when it’s surrounded by others of the same species, size, and apparent health with few SLF, suggesting that some trees are better hosts than others (17). Larger sample sizes may have mitigated high variability, but sample size was limited by the time required to take measurements with the LiCor instrument during peak solar radiation. In some cases, the duration of the experiments may have been too short to impact the plant metrics we measured, but it was not possible to maintain SLF at the same life stage and density beyond the duration of our experiments, introducing a confounding factor we could not control.

We suggest that impacts of SLF in the wild are greater when trees are also stressed by other biotic or abiotic factors; under these circumstances SLF is another stressor and effects can be cumulative. In 2019, we observed poor health and structural damage of red maples with cankers at the union of main branches after being heavily fed on during the 2018 growing season when rainfall was 150% of normal in Pennsylvania (35). Cultures were taken from these cankers, but only opportunistic, endemic fungi were detected (*Botryosphaeria* and *Nectria* spp.), which are usually benign, but spores can invade trees through wounds when splashed around by rain (36).

In summary, our results suggest that SLF late-stage nymphs feeding at high densities for relatively short durations of time on young maples may have only minor effects on gas exchange attributes, but could significantly reduce nutrient concentrations such as carbohydrates and nitrogen, which in turn may reduce diameter growth. Tree of heaven was more affected by adults than silver maple trees of similar size, suggesting that higher numbers may overwhelm the defenses of tree of heaven, which co-evolved with SLF in its native range (9). This finding also helps explain our observations of eventual death of saplings and mature tree of heaven. At the same time, declines in C assimilation in response to adult feeding in tree of heaven were not reflected in altered nutrient concentrations in roots or leaves, in contrast to the marked impacts on below-ground C and N content in young grapevines in response to heavy adult feeding pressure (5).

Based on our results, we recommend that production nurseries, forest managers, and homeowners continue to protect young maple and black walnut saplings, especially once SLF become adults, and minimize plant stressors to mitigate cumulative impacts. In the wild, we observe that SLF are more likely to feed on larger trees as they develop into later life stages, and more mature trees may not experience significant harm from even high populations, although late season feeding can occur for a month or more on maples for multiple years (17). Moreover, if trees are stressed, we cannot rule out that even larger trees may suffer reduced health and growth given that no long-term studies have been done on mature trees in response to SLF feeding.

## Data availability statement

The original datasets used for this study can be found in Penn State University’s Scholar Sphere at https://scholarsphere.psu.edu/resources/c0418d6c-6b38-407d-b879-469fffc6d442/ and in the **Supplementary Materials**. Further inquiries can be directed to the corresponding author.

## Author contributions

DE and KH funded the study and planned the experimental design with EL. EL conducted most of the experiments, assisted by EP, OU, JH, and LI. LI conducted the data analyses and prepared the tables and figures. LI and KH wrote the manuscript. BW designed and planted the common garden and consulted on planning experiments. All authors contributed to the article and approved the submitted version.
