# Supplementary material for: Impacts of short-term feeding by spotted lanternfly (Lycorma delicatula) on ecophysiology of young hardwood trees in a common garden
Source: Front Insect Sci. 2022 Dec 7;2:1080124. doi: 10.3389/finsc.2022.1080124 (PMC10926534; doi:10.3389/finsc.2022.1080124)
Supplement: Supplementary file 1 [file DataSheet_1.docx]

**Supplemental Information**

**Table S1**. Details of experiments conducted with different densities of SLF in sleeve cages or whole tree enclosures in 2019 and 2021 on red maple (*Acer rubrum)*, silver maple *(Acer saccharinum)*, black walnut *(Juglans nigra)* and tree of heaven *(Ailanthus altissima)*. Notes: date format as month/day/year.

| **Experiment setup & data collected** |  | | | |
| --- | --- | --- | --- | --- |
|  | **2019** | | **2020** | |
| SLF enclosure type | **sleeve cages** | **sleeve cages** | **whole-tree enclosures** | **whole-tree enclosures** |
| SLF life stage | 4^th^ instars | adults | 4^th^ instars | adults |
| SLF treatment density per enclosure | 0, 15, 30 | 0, 40 | 0, 40, 80, 120 | 0, 40, 80, 120 |
| Experiment duration and dates | 12 days (July 22 - August 2) | 11 days (September 24 - October 4) | 10 days (July 22 – 31) | 20 days (August 6 – 25) |
| Tree species | silver maple & red maple | silver maple & red maple | silver maple & black walnut | silver maple & tree of heaven |
| Experimental structure | 2 tree species x 3 SLF densities x 6 replicates per treatment combination =   36 experimental trees | 2 tree species x 2 SLF densities x 10 replicates per treatment combination = 40 experimental trees | 2 species x 4 SLF densities x 5 replicates per treatment combination = 40 experimental trees | 2 tree species x 4 SLF densities x 5 replicates per treatment combination = 40 experimental trees |
| Gas exchange measurement frequency and dates | 2 times (7/18, 8/2) | 4 times (9/24, 9/27, 10/1, 10/4) | 4 times (7/22, 7/26, 7/29, 7/31) | 7 times (8/6, 8/9, 8/12, 8/14, 8/17, 8/20, 8/25) |
| Carbohydrate content: tissue type and collection dates | *NT | bark (10/4; 3/27/2020), wood (10/4; 3/27/2020), roots (2/11/2020) | branch (7/31; 4/7/2021), leaves (7/22, 7/31) | branch (8/25; 4/7/2021), leaves (8/25), roots (4/7/2021) |
| Nitrogen content: tissue type and collection dates | NT | leaves (10/1/2019) | NT | leaves (8/25/2020); roots (4/7/2021) |
| Tree growth metrics recorded | NT | NT | DBH increase recorded after 2020 and 2021 growing seasons (4/7/2021, 3/16/2022) | DBH increase recorded after 2020 and 2021 growing seasons (4/7/2021, 3/16/2022) |

*NT = not tested or not measured

**Table S2**. Fixed and random effects for best fit mixed model estimating the rate of carbon assimilation (response variable was natural log transformed as ln [rate+1]) during the experiment with *4^th^ instar* SLF in sleeve cages on red maple and silver maple measured on August 2, 2019 after 12 days of feeding pressure (*n* = 36). Notes: RM = red maple*.* “SLF” variable denotes different densities of SLF treatment (0, 15, and 30 SLF per sleeve cage). SE = standard error, model *p*-values < 0.05 are highlighted in bold font.

| **Variable** | **Model parameter estimates (SE)** | ***P*-value** |
| --- | --- | --- |
| **Intercept** | 2.0742 (0.0874) | **<0.0001** |
| **Tree Species [RM]** | -0.0317 (0.0874) | 0.7244 |
| **SLF [0]** | 0.0214 (0.0924) | 0.8187 |
| **SLF [15]** | -0.2559 (0.0924) | **0.0112** |
| **Tree number (random)** | 0.0403 (0.0438) | 0.3567 |
| **Overall model AIC/BIC** | 52/ 59 | |

**Table S3a.** Fixed and random effects for best generalized linear mixed model (with a gamma distribution and a Log link function) estimating carbon assimilation measured during the experiment with *adults* SLF in sleeve cages in 2019. Notes: RM = red maple. “Day” variable denotes separate days of measurement since the start of the experiment. Gas exchange was measured on Sept. 24 and 27, Oct. 1, and Oct. 4, 2019 (*n* = 86). “SLF” variable denotes different densities of SLF treatment (0 and 40 SLF per sleeve cage). SE = standard error. Model *p*-values < 0.05 are highlighted in bold font.

| **Variables** | **Model parameter estimates (SE)** | ***P*-value** |
| --- | --- | --- |
| **Intercept** | 2.8166 (0.3859) | **<0.0001** |
| **Tree species [RM]** | -0.2198 (0.2186) | 0.3289 |
| **Day of experiment** | -0.2191 (0.0555) | **0.0002** |
| **SLF [0]** | 1.1156 (0.1909) | **<0.0001** |
| **Tree number (random effect)** | 0.2143(0.2760) | 0.4374 |
| **Overall model AIC/ BIC** | 357 / 371 | |

**Table S3b.** Results of multiple comparisons test (Tukey HSD post-hoc test) to determine if the sleeve cages alone affected average (least square means) carbon assimilation rates (µmol m^-2^ s^-1^) during the experiment with *adult* SLF in sleeve cages in 2019. Notes: SM = silver maple, RM = red maple. Each tree type (control or treated) had a branch with no sleeve, a branch with a sleeve but no SLF (to test the effect of sleeve alone), or a branch with a sleeve that confined 40 adult SLF for treated trees only. No significant differences (*p* < 0.05) were found between branches with and without sleeves for control or treated trees. Gas exchange was measured on Sept. 24 and 27, Oct. 1, and Oct. 4, 2019 (*n* = 173).

| **Days since SLF infestation** | **1** | | **3** | | **7** | | **10** | |
| --- | --- | --- | --- | --- | --- | --- | --- | --- |
| **Tree Species** | **RM** | **SM** | **RM** | **SM** | **RM** | **SM** | **RM** | **SM** |
| **Branch type** |  | | | | | | | |
| **Control – no sleeve** | 10.40 | 15.35 | 11.56 | 14.15 | 9.58 | 17.25 | 6.00 | 14.42 |
| **Control – sleeve** | 10.62 | 15.42 | 14.35 | 16.05 | 9.19 | 18.13 | 8.39 | 12.31 |
| **Treatment – no sleeve** | 7.91 | 14.98 | 8.66 | 19.97 | 7.86 | 17.63 | 7.09 | 15.33 |
| **Treatment – sleeve, no SLF** | 7.34 | 15.64 | 8.05 | 15.13 | 7.51 | 14.84 | 7.39 | 11.53 |

**Table S4A and B**. Fixed and random effects for best fit mixed model estimating gas exchange attributes (response variable was natural log transformed as ln [rate+1]) or a generalized linear mixed model (GLMM, with a gamma distribution and a Log link function) measured during whole-tree enclosures experiments with (A) *4^th^ instar (n* = 155*)* or (B) *adult* (*n* = 270) SLF in 2020. SM = silver maple, BW = black walnut. “SLF” variable denotes different densities of feeding pressure (0, 40, 80, and 120 SLF per enclosure). “Day” variable denotes separate days of measurement since the start of the experiment. Gas exchange was measured on July 22, 26, 29, and 31 for the nymph experiment, and on August 6, 9, 12, 14, 17, 20, and 25 for the adult experiment. “Day*Day” variable tests if there was a quadratic relationship over time. SE = standard error. Model *p*-values < 0.05 (statistically significant) are in bolded black font and *p*-values < 0.1 (marginally significant) are in red font. N/A indicates factors that were not present in best fit model.

1. **4^th^ instars**

| **Variables** | **C assimilation** | | **Transpiration** | | **Conductance** | |
| --- | --- | --- | --- | --- | --- | --- |
|  | **Model parameter estimates (SE)** | ***P*- values** | **Model parameter estimates (SE)** | ***P*- values** | **Model parameter estimates (SE)** | ***P*- values** |
| **Intercept** | 2.4250 (0.0947) | **<0.0001** | 1.4949 (0.0759) | **<0.0001** | 0.1257 (0.0207) | **<0.0001** |
| **SLF [0]** | -0.0267 (0.1120) | 0.8128 | 0.0662 (0.0092) | 0.4692 | 0.0223 (0.0270) | 0.4158 |
| **SLF [40]** | 0.0047 (0.1111) | 0.9662 | -0.0036 (0.0894) | 0.9682 | -0.0069 (0.0269) | 0.7981 |
| **SLF [80]** | 0.0305 (0.1116) | 0.7859 | 0.0203 (0.0897) | 0.8227 | 0.0049 (0.0270) | 0.8563 |
| **Tree species [BW]** | 0.1594 (0.0644) | **0.0183** | 0.4052 (0.0519) | **<0.0001** | 0.1292 (0.0156) | **<0.0001** |
| **Day** | 0.0171 (0.0115) | 0.1386 | 0.0074 (0.00092) | 0.4199 | 0.0169 (0.0031) | **<0.0001** |
| **Day * Day** | N/A | N/A | N/A | N/A | 0.0039 (0.0011) | **0.0004** |
| **Tree number (random)** | 0.1062 (0.0400) | **0.0080** | 0.0691 (0.0273) | **0.0113** | 0.0061 (0.0023) | **0.0095** |
| **Overall model AIC / BIC** | 264 / 288 | | 194 / 217 | | -171 / -145 | |

1. **Adults**

| **Variables** | **Carbon assimilation**  GLMM - Gamma (Log link function) | | **Transpiration**  GLMM - Gamma (Log link function) | | **Conductance** | |
| --- | --- | --- | --- | --- | --- | --- |
|  | **Model parameter estimates (SE)** | ***P*- values** | **Model parameter estimates (SE)** | ***P*- values** | **Model parameter estimates (SE)** | ***P*- values** |
| **Intercept** | 2.8952 (0.0411) | **<0.0001** | 1.6373 (0.0522) | **<0.0001** | 0.4415 (0.0138) | **<0.0001** |
| **SLF [0]** | 0.1563 (0.0404) | **0.0005** | 0.1905 (0.0605) | **0.0034** | 0.0525 (0.0170) | **0.0040** |
| **SLF [40]** | 0.0817 (0.0388) | **0.0422** | 0.0975 (0.0575) | **0.0984** | 0.0136 (0.0161) | 0.4028 |
| **SLF [80]** | -0.2146 (0.0389) | **<0.0001** | -0.2357 (0.0582) | **0.0003** | -0.0577 (0.0163) | **0.0011** |
| **Tree species [SM]** | -0.0367 (0.0227) | **<0.0001** | -0.5878 (0.0340) | **<0.0001** | -0.01851 (0.0095) | **<0.0001** |
| **Day** | -0.0232 (0.0404) | **<0.0001** |  |  | -0.0046 (0.0010) | **<0.0001** |
| **Day * Species [SM]** | N/A | N/A | N/A | N/A | 0.0041 (0.001) | **<0.0001** |
| **Day * SLF [0]** | N/A | N/A | N/A | N/A | 0.0014 (0.0018) | 0.4444 |
| **Day * SLF [40]** | N/A | N/A | N/A | N/A | 0.0036 (0.0017) | **0.0360** |
| **Day * SLF [80]** | N/A | N/A | N/A | N/A | -0.0074 (0.0017) | **<0.0001** |
| **Day * Species [SM] * SLF [0]** | N/A | N/A | N/A | N/A | -0.0042 (0.0018) | **0.0223** |
| **Day * Species [SM] * SLF [40]** | N/A | N/A | N/A | N/A | -0.0038 (0.0017) | **0.0276** |
| **Day * Species [SM] * SLF [80]** | N/A | N/A | N/A | N/A | 0.0095 (0.0017) | **<0.0001** |
| **Tree number (random)** | 0.0029 (0.0051) | 0.5728 | 0.0222 (0.0111) | **0.0449** | 0.0021 (0.0009) | **0.0159** |
| **Overall model AIC/BIC** | 242 / 270 | | 335 / 363 | | -425 / -372 | |

**Table S5a**. Fixed effects for best fit mixed model estimating non-structural carbohydrate concentrations (response variable was natural log transformed as ln [concentration+1]) measured in root, or branch wood and bark tissues of silver maple and red maple trees exposed to *adult* SLF in sleeve cages for 10 days in 2019. “SLF” variable denotes different densities of feeding pressure (0 or 40 SLF per enclosure). RM = red maple, SE = standard error. Roots samples (n = 20) were collected on February 11, 2020, wood and bark samples were collected on October 4, 2019 (fall sampling, n = 39 bark, n = 30 wood), and March 27, 2020 (spring sampling, n = 40 bark, n = 40 wood). Model *p*-values < 0.05 are highlighted in bold font. N/A indicates factors that were not present in best fit model.

| **Variables** | **Roots,**  **Fraction of soluble** | | **Wood, Fall sampling,**  **Soluble** | | **Wood, Spring sampling,**  **Soluble** | | **Bark, Fall sampling,**  **Fraction**  **of soluble** | | **Bark, Spring sampling,**  **Fraction**  **of soluble** | |
| --- | --- | --- | --- | --- | --- | --- | --- | --- | --- | --- |
|  | **Model parameter estimates (SE)** | ***P*- values** | **Model parameter estimates (SE)** | ***P*- values** | **Model parameter estimates (SE)** | ***P*- values** | **Model parameter estimates (SE)** | ***P*- values** | **Model parameter estimates (SE)** | ***P*- values** |
| **Intercept** | 0.3946 (0.0306) | **<0.0001** | 1.8732 (0.0784) | **<0.0001** | 2.0382 (0.0590) | **<0.0001** | 0.4653 (0.0119) | **<0.0001** | 0.5319 (0.0133) | **<0.0001** |
| **SLF [0]** | 0.0145 (0.0306) | 0.9432 | 0.2071 (0.0784) | **0.0138** | -0.0923 (0.0591) | 0.1267 | -0.0075 (0.0119) | 0.5328 | 0.0045 (0.0133) | 0.7363 |
| **Tree species [RM]** | -0.0022 (0.0307) | 0.6415 | -0.0571 (0.0784) | 0.4729 | 0.1287 (0.0591) | **0.0358** | -0.0180 (0.0119) | 0.1378 | 0.0521 (0.0133) | **0.0004** |
| **Species x SLF** | N/A | N/A | -0.2649 (0.0784) | **0.0023** | 0.3225 (0.0590) | **<0.0001** | N/A | N/A | N/A | N/A |
| **Overall model AIC / BIC** | -15 / -14 | | 39 / 43 | | 42 / 49 | | -86 / -80 | | -78 / -72 | |

**Table S5b****.** Results of multiple comparisons test (Tukey HSD post-hoc test) to determine SLF treatment effects on non-structural carbohydrate concentrations (mg g^-1^) measured in branch wood tissues of silver maple and red maple trees exposed to *adult* SLF in sleeve cages for 10 days in 2019. Different densities of SLF feeding pressure were 0 or 40 SLF per enclosure. Wood samples were collected on October 4, 2019 (fall sampling, *n* = 30), and March 27, 2020 (spring sampling, *n* = 40). Carbohydrate concentrations are given in mg of glucose equivalents per g of dry tissue. Significant (*p* < 0.05) differences in concentrations between treatments for a given carbohydrate type within a sampling season are denoted with different letters after the mean.

| **Tree species / SLF density** | **Fall** | | | **Spring** | | |
| --- | --- | --- | --- | --- | --- | --- |
|  | **Soluble sugar** | **Starch** | **Total non-structural carbohydrates** | **Soluble sugar** | **Starch** | **Total non-structural carbohydrates** |
| **Silver maple** |  | | |  | | |
| **0** | 10.36 ^a^ | 17.97 ^a^ | 28.33 ^a^ | 3.45 ^c^ | 16.16 ^a^ | 19.62 ^a^ |
| **40** | 3.65 ^b^ | 15.78 ^a^ | 19.43 ^a^ | 9.21 ^ab^ | 10.80 ^a^ | 20.02 ^a^ |
| **Red maple** |  | | |  | | |
| **0** | 5.11 ^b^ | 15.72 ^a^ | 20.83 ^a^ | 9.99 ^a^ | 8.01 ^a^ | 18.00 ^a^ |
| **40** | 6.23 ^ab^ | 14.87 ^a^ | 21.10 ^a^ | 5.93 ^bc^ | 12.78 ^a^ | 18.72 ^a^ |

**Table S6a.** Fixed effects for best fit mixed model estimating non-structural carbohydrate concentrations (response variable was natural log transformed as ln [concentration+1]) or a generalized linear mixed model (GLMM, with a gamma distribution and a Log link function) measured in branch and leaf tissue of silver maple and black walnut exposed to *4^th^ instar nymph* SLF feeding pressure (0, 40, 80, or 120 SLF) in whole tree enclosures in 2020 for 10 days. “SLF” variable denotes different densities of SLF feeding pressure. SM = silver maple, SE = standard error. Branch samples were collected on July 31, 2020 (summer sampling, *n* = 39), and April 7, 2021 (spring sampling, *n* = 27), and leaf samples (*n* = 73) were collected on July 22 and 31, 2020. Note: the overall model shows statistically significant “SLF x Species” variable, but the effects of SLF treatments were different only for silver maple, and not different for black walnut when compared to controls (Tukey post-hoc pairwise comparison, data not shown). Model *p*-values < 0.05 (considered statistically significant) are in bolded black font and *p*-values < 0.1 (considered marginally significant) are in red font. N/A indicates a factor that was not present in the best fit model.

| **Variables** | **Branch, Summer sampling, Soluble**  GLMM - Gamma (Log link function) | | **Branch, Spring sampling,**  **Soluble** | | **Leaves,**  **Fraction soluble**  GLMM - Gamma (Log link function) | |
| --- | --- | --- | --- | --- | --- | --- |
|  | **Model parameter estimates (SE)** | ***P*- values** | **Model parameter estimates (SE)** | ***P*- values** | **Model parameter estimates (SE)** | ***P*- values** |
| **Intercept** | 3.4706 (0.0383) | **<0.0001** | 2.5835 (0.0434) | **<0.0001** | -0.2696 (0.0192) | **<0.0001** |
| **SLF [0]** | 0.1883 (0.0658) | **0.0072** | 0.0295 (0.0774) | 0.7050 | 0.0433 (0.0331) | 0.1995 |
| **SLF [40]** | 0.1142 (0.0658) | **0.0921** | -0.1372 (0.0749) | **0.0759** | 0.0017 (0.0326) | 0.9597 |
| **SLF [80]** | -0.0751 (0.0658) | 0.2622 | 0.0948 (0.0745) | 0.2121 | -0.0936 (0.0334) | **0.0081** |
| **Tree species** | 0.3650 (0.0383) | **<0.0001** | [SM] 0.1834 (0.0436) | **0.0002** | 0.1476 (0.0191) | **<0.0001** |
| **Species [BW] x SLF [0]** | N/A | N/A | N/A | N/A | -0.0267 (0.0331) | 0.4256 |
| **Species [BW] x SLF [40]** | N/A | N/A | N/A | N/A | -0.0368 (0.0326) | 0.2664 |
| **Species [BW] x SLF [80]** | N/A | N/A | N/A | N/A | 0.1367 (0.0333) | **0.0002** |
| **Day of experiment** | N/A | N/A | N/A | N/A | -0.0881 (0.01771) | **<0.0001** |
| **Overall model AIC / BIC** | 32 / 40 | | 18 / 25 | | 2 / 23 | |

**Table S6b.** Results of multiple comparisons test (Tukey HSD post-hoc test) to determine SLF treatment effects on non-structural carbohydrate concentrations (mg g^-1^) measured in branch and leaf tissue of silver maple exposed to *4^th^ instar nymph* SLF feeding pressure (0, 40, 80, or 120 nymphs) in whole tree enclosures in 2020 for 10 days. Branch samples (*n* = 39) were collected on July 31, 2020, and leaf samples (*n* = 73) were collected on July 22 and 31, 2020. Carbohydrate concentrations are given in mg of glucose equivalents per g of dry tissue. Fraction of soluble to total non-structural carbohydrates is expressed as a value between 0 and 1. Significant (*p* ≤ 0.05) differences in concentrations between treatments for a given carbohydrate type within a tissue type are denoted with different letters. Treatment means significantly different from control (*p* < 0.05) highlighted in bold font.

| **Tissue type / SLF density** | **Carbohydrate type** | | | |
| --- | --- | --- | --- | --- |
|  | **Soluble sugar** | **Starch** | **Total non-structural carbohydrates** | **Fraction of soluble to total sugars** |
| **Leaves** |  | | | |
| **0** | 53.93 ^a^ | 21.49 ^a^ | 75.43 ^a^ | 0.71 ^a^ |
| **40** | 57.85 ^a^ | 22.64 ^a^ | 80.49 ^a^ | 0.69 ^a^ |
| **80** | 47.71 ^a^ | 41.60 ^a^ | 89.31 ^a^ | 0.53 ^a^ |
| **120** | 57.16 ^a^ | 18.44 ^a^ | 75.60 ^a^ | **0.75 ^b^** |
| **Branch** |  | | | |
| **0** | 30.18 ^abc^ | 6.30 ^a^ | 36.49 ^ab^ | 0.77 ^a^ |
| **40** | 27.20 ^bc^ | 9.00 ^a^ | 36.21 ^ab^ | 0.71 ^a^ |
| **80** | 18.21 ^cd^ | 7.67 ^a^ | 25.88 ^bc^ | 0.69 ^a^ |
| **120** | **14.18 ^d^** | 5.50 ^a^ | **19.69 ^c^** | 0.67 ^a^ |

**Table S7**. Fixed effects for best fit mixed model estimating non-structural carbohydrate concentrations (response variable was natural log transformed as ln [concentration+1]) measured in root and branch tissue of silver maple and tree of heaven exposed to *adult SLF* in whole tree enclosures in 2020 for 20 days. “SLF” variable denotes different densities of SLF feeding pressure (0, 40, 80, or 120 SLF per enclosure). SM = silver maple, SE = standard error. Roots samples (*n* = 39) were collected on April 4 and 11, 2021, leaf samples (*n* = 38) on August 25, 2020, and branch samples on August 25, 2020 (summer sampling, *n* = 34), and on April 7, 2021 (spring sampling, *n* = 39). Note: the overall model shows statistically significant “SLF” for spring branch, but the effects of SLF treatments were different only between species, and not different within species when compared to controls (Tukey post-hoc pairwise comparison, data not shown). Model *p*-values < 0.05 (considered statistically significant) are in bolded black font and *p*-values < 0.1 (considered marginally significant) are in red font.

| **Variables** | **Roots,**  **Soluble** | | **Leaves,**  **Soluble** | | **Branch, Summer sampling,**  **Soluble** | | **Branch, Spring sampling**  **Soluble** | |
| --- | --- | --- | --- | --- | --- | --- | --- | --- |
|  | **Model parameter estimates (SE)** | ***P*- values** | **Model parameter estimates (SE)** | ***P*- values** | **Model parameter estimates (SE)** | ***P*- values** | **Model parameter estimates (SE)** | ***P*- values** |
| **Intercept** | 2.3909 (0.0959) | **<0.0001** | 4.5378 (0.0398) | **<0.0001** | 3.6291 (0.0499) | **<0.0001** | 2.5832 (0.0434) | **<0.0001** |
| **SLF [0]** | -0.1644 (0.1717) | 0.3424 | -0.0507 (000533) | 0.3527 | -0.1130 (0.0847) | 0.1928 | 0.0295 (0.0774) | 0.7050 |
| **SLF [40]** | -0.0513 (0.1646) | 0.7571 | -0.0396 (0.0509) | 0.4435 | 0.0632 (0.0881) | 0.4788 | -0.1371 (0.0749) | **0.0759** |
| **SLF [80]** | -0.0513 (0.1646) | 0.4115 | 0.0482 (0.0522) | 0.3670 | 0.0619 (0.0881) | 0.4879 | 0.0948 (0.0745) | 0.2121 |
| **Tree species [SM]** | -0.0041 (0.0959) | 0.9660 | -0.2185 (0.0250) | **<0.0001** | 0.0627 (0.0499) | 0.1986 | 0.1834 (0.0436) | **0.0002** |
| **Overall model AIC / BIC** | -80 / 87 | | -25 / -17 | | 22 / 28 | | 18 / 25 | |

**Table S8.** Fixed effects for best fit mixed model estimating nitrogen levels (response variable was natural log transformed as ln [concentration+1]) measured in leaf tissue of silver maple and red maple trees exposed to *adult SLF* in sleeve cages in 2019 for 10 days. “SLF” variable denotes different densities of SLF feeding pressure (0 or 40 SLF per enclosure). RM = red maple. SE = standard error. Leaf samples (*n* = 12) were collected on October 1, 2019. Model *p*-values < 0.05 (considered statistically significant) are in bolded black font and *p-*values < 0.1 (considered marginally significant) are in red font.

| **Variables** | **Model parameter estimates (SE)** | ***P*- values** |
| --- | --- | --- |
| **Intercept** | 1.2602 (0.0264) | **<0.0001** |
| **SLF [0]** | 0.1037 (0.0167) | **0.0914** |
| **Tree species [RM]** | -0.1121 (0.0224) | **0.0012** |
| **Overall model AIC / BIC** | -14 / -22 | |

**Table S9**. Fixed effects for best fit mixed model estimating nitrogen levels (response variable was natural log transformed as ln [concentration+1]) or a generalized linear mixed model (GLMM, with a gamma distribution and a Log link function) measured in root and leaf tissues of silver maple and tree of heaven exposed to *adult SLF* in whole tree enclosures at the common garden in 2020 for 10 days. “SLF” variable denotes different densities of SLF treatment (0, 40, 80, or 120 SLF per enclosure). SM = silver maple, SE = standard error. Roots samples (*n* = 39) were collected on April 7, 2021 and leaf samples (*n* = 38) were collected on August 25, 2020. Note: the overall model shows statistically significant “SLF*Species” and “SLF” for leaves, but the effects of SLF treatments were different only between species, and not different within species when compared to controls (Tukey and Games-Howell post-hoc pairwise comparison tests, data not shown). Model *p*-values < 0.05 are highlighted in bold font.

| **Variables** | **Roots** | | **Leaves**  GLMM - Gamma (Log link function) | |
| --- | --- | --- | --- | --- |
|  | **Model parameter estimates (SE)** | ***P*- values** | **Model parameter estimates (SE)** | ***P*- values** |
| **Intercept** | 0.08252 (0.0219) | **<0.0001** | 0.9232 (0.0202) | **<0.0001** |
| **SLF [0]** | -0.0551 (0.0386) | 0.1643 | 0.0148 (0.0355) | 0.6786 |
| **SLF [40]** | 0.0370 (0.0386) | 0.3462 | 0.0388 (0.0343) | 0.2660 |
| **SLF [80]** | -0.0488 (0.0372) | 0.1993 | -0.0720 (0.0343) | **0.0436** |
| **Tree species [SM]** | 0.0864 (0.0219) | **0.0005** | -0.0934 (0.0202) | **<0.0001** |
| **Overall model AIC / BIC** | -33 / -27 | | -41 / -34 | |

**Figure S1**. Average (least square means) C assimilation (µmol m^-2^ s^-1^) during the experiment with *4^th^ instar* SLF in sleeve cages on red maple and silver maple measured on August 2, 2019 after 12 days of feeding pressure (*n* = 36). Significant differences (p < 0.05) between treatments are shown as different letters above the standard error bars.

**
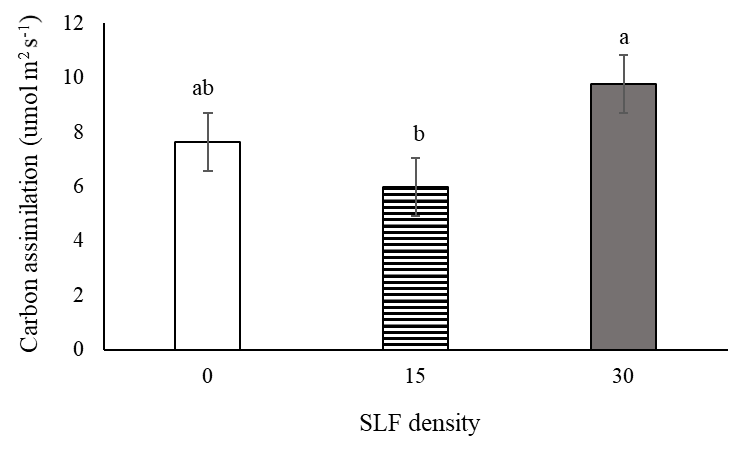
**

**Figure S2**. Carbon assimilation over time for black walnut (A) and silver maple (B) in 2020. Spotted lanternfly *4^th^ instar* densities were 0, 40, 80, or 120 inside whole-tree enclosures. Gas exchange was measured on July 22, 26, 29, and 31. Note the differences in carbon assimilation rates on the y-axes. There were no significant differences between treatments at any time point.


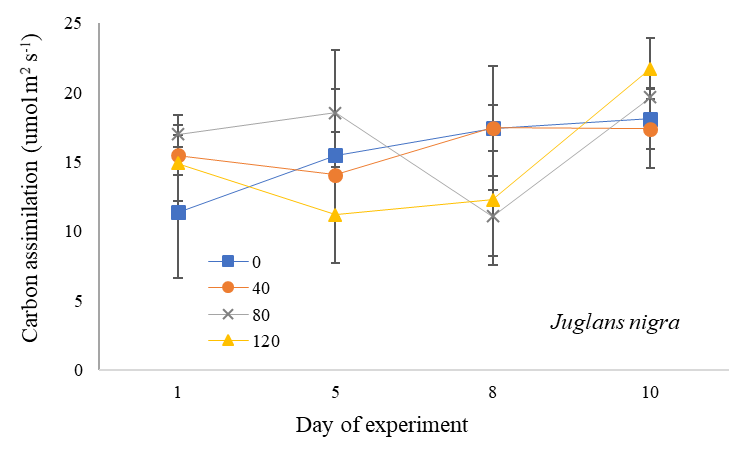


(A)


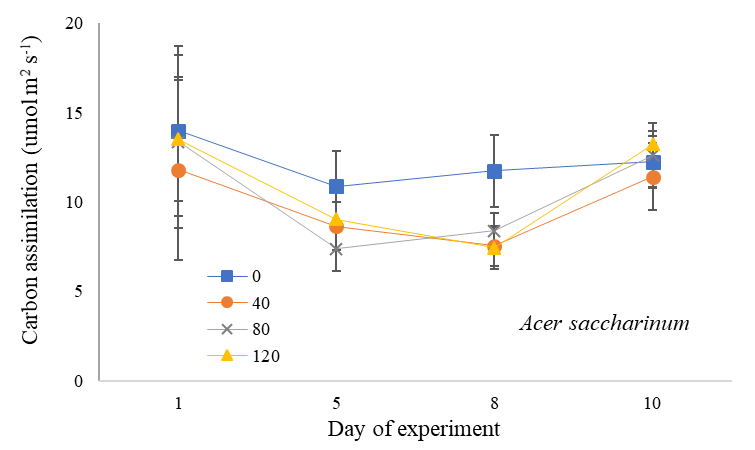


(B)

**Figure S3**. Stomatal conductance over time for tree of heaven (A) and silver maple (B) in 2020 with SLF *adults* confined by whole tree enclosures at one of four densities (0, 40, 80, and 120). Gas exchange was measured on August 6, 9, 12, 14, 17, 20, and 25. Note the differences in conductance rates on the y-axes. Asterisks above the standard error bars denote dates when conductance on treatment trees were marginally different from controls (*p* < 0.1).


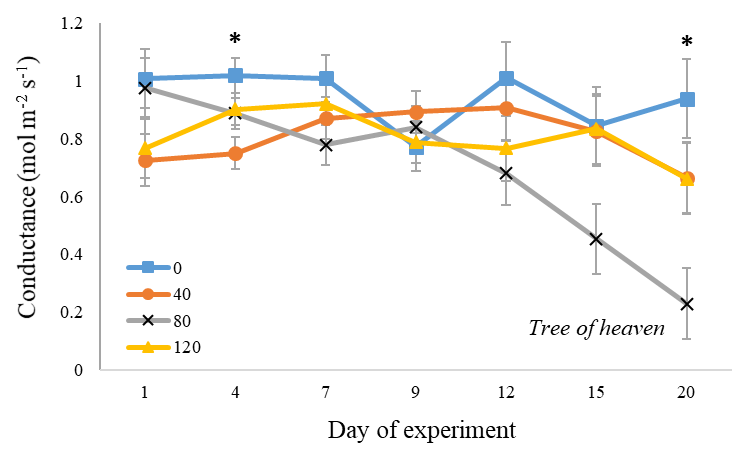


(A)

(B)

**
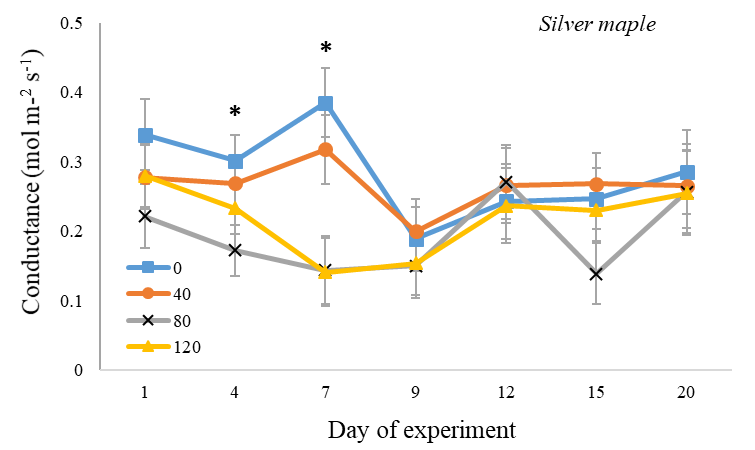
**

**Figure S4**. Transpiration over time for tree of heaven (A) and silver maple (B) with SLF *adult*s confined by whole tree enclosures for 10 days at one of four densities (0, 40, 80, or 120). Gas exchange was measured on August 6, 9, 12, 14, 17, 20, and 25. Note the differences in transpiration rates on the y-axes. Asterisks above the standard error bars denote dates when transpiration on treatment trees was marginally different from controls (*p* < 0.1).


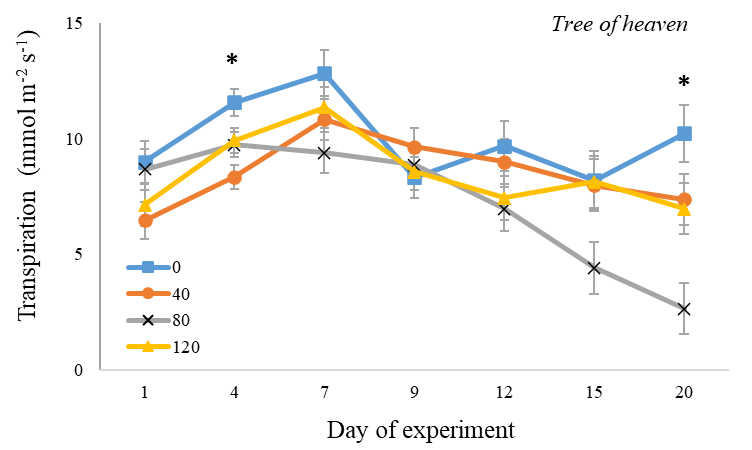


(A)


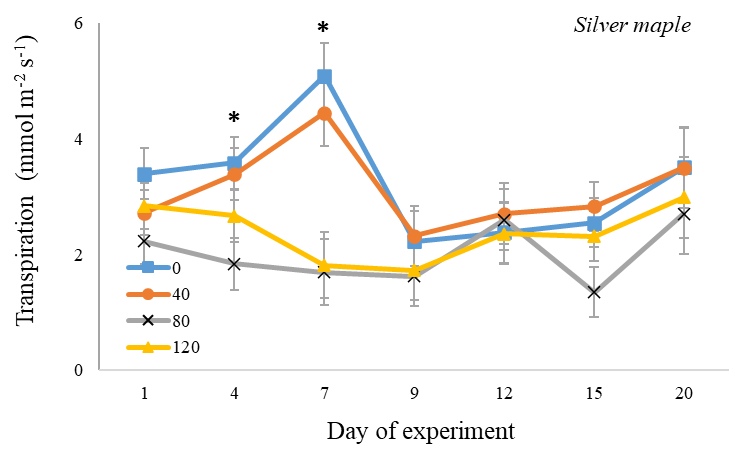


(B)
